# Clonality, spatial structure, and pathogenic variation in *Fusarium fujikuroi* from rain-fed rice in southern Laos

**DOI:** 10.1371/journal.pone.0226556

**Published:** 2019-12-23

**Authors:** Barbara Scherm, Virgilio Balmas, Alessandro Infantino, Maria Aragona, Maria Teresa Valente, Francesca Desiderio, Angela Marcello, Sengphet Phanthavong, Lester W. Burgess, Domenico Rau

**Affiliations:** 1 Dipartimento di Agraria, Sezione di Patologia ed Entomologia, Università degli Studi di Sassari, Sassari, Italy; 2 Council for Agricultural Research and Economics (CREA), Research Centre for Plant Protection and Certification, Rome, Italy; 3 Council for Agricultural Research and Economics, Research Centre for Genomics and Bioinformatics, Fiorenzuola d’Arda (PC), Italy; 4 Provincial Agriculture and Forestry, Thaluang Village, Pakse, Champasak, Lao PDR; 5 Sydney Insitute of Agriculture, Faculty of Science, University of Sydney, New South Wales, Australia; 6 Dipartimento di Agraria, Sezione di Patologia ed Entomologia, Università degli Studi di Sassari, Sassari, Italy; Universita degli Studi di Pisa, ITALY

## Abstract

Bakanae disease, caused by the fungal phytopathogen *Fusarium fujikuroi*, can be detected in most rice (*Oryza sativa* L.) growing areas worldwide. In this study, we investigated the population structure of this fungus in southern Lao PDR, a country located near the geographic origin of rice domestication. Microsatellites (SSRs) and mating type (MAT) analyses, pathogenicity and fungicide sensitivity tests were integrated in the study. The first key finding is that the population genetic structure of *F*. *fujikuroi* in Lao PDR is consistent with high clonal reproduction. Indeed, (i) “true” clones were identified; (ii) within populations, MAT types were frequently skewed from 1:1 ratio, (iii) linkage disequilibrium (among SSRs as also among SSRs and MAT) was present, and (iv) gene-flow between opposite MAT types within the same population is restricted. The presence of genetic divergence among areas and populations and the occurrence of positive spatial autocorrelation of genetic variation, indicate that migration is restricted, and that genetic drift plays an important role in the evolution of this fungus. Two main well-defined groups of isolates were detected (F_ST_ = 0.213) that display a non-random spatial distribution. They differ in the ability to induce seedlings death but not seedlings elongation (the typical Bakanae symptom) suggesting that the pathogen’s ability to induce the two symptoms is under different genetic control. Finally, we compared two agroecosystems with contrasting characteristics: low-input and traditional (Lao PDR) vs high-input and modern (Italy). We found differences in the level of population structuring and of spatial autocorrelation. This suggests that the evolutionary potential of the fungus not only depends on its intrinsic characteristics, but is strongly influenced by other external factors, most likely by the dynamics of infested seed exchange. Thus, quarantine and chemical treatments are a way to reduce population connectivity and hence the evolutionary potential of this pathogen.

## Introduction

Bakanae disease, caused by the fungal phytopathogen *Fusarium fujikuroi*, Nirenberg, is present in most rice (*Oryza sativa* L.) growing areas worldwide [1, 2, 3]. Moreover, *F*. *fujikuroi* is considered the cause of dry-rot, brown lesions and necrotic leaf spot on pineapple [4], as well as pre- and post-emergence damping-off on soybean [5]. However, when these isolates are artificially inoculated onto their respective hosts and rice, stem elongation was observed only in rice seedlings [5]. *F*. *fujikuroi* has also been reported in several other crops (e.g., maize, wheat, barley, rye, sugarcane, sorghum, asparagus, vanilla, dragon fruit and *Canna edulis* [6, 7, 8, 9]. Several other crops can act as alternative hosts [6]. *F*. *fujikuroi* has also been isolated from human skin in Iowa (USA) [10].

The name Bakanae means “absurd plant” or “foolish seedlings” in Japanese and derives from the abnormal stem elongation in the infected rice plant due to the production of gibberellic acid (GA3) by the fungus. Other symptoms may be present on affected plants such as pale green flag leaves and later, sterile empty panicles. Whitish-pinkish mycelium and pink sporodochia of the fungus can be observed on the infected stem. If the pathogen is present in the early stage of plant growth, it can cause seedling death or induce browning and stem rot in the crown region. In fact, greenhouse experiments showed that following artificial inoculation, the same isolate can induce both seedling blight or seedling elongation [11]. The fungus can form macro- and microconidia on the infected stem. These conidia can be disseminated by wind or water splash and infect the inflorescence of developing panicles of rice plants, leading to contamination of the seeds. Furthermore, the fungus is capable of both asexual and sexual reproduction and both conidia and ascospores can play relevant roles in the disease cycle [1]. *Fusarium fujikuroi* is generally transmitted through infected seed, but it can also survive in the soil in infected crop residues that represent an additional source of inoculum.

Rice is one of the most important and ancient food crops in the world [12]. It is cultivated in over 163 million ha in more than 100 countries (http://www.fao.org/faostat/en/#home).

In 2017, rice was cultivated on 956,134 ha in the Lao PDR with a total production of 4,039,779 tonnes (http://www.fao.org/faostat/en/#data/QC). Rain-fed rice is a key staple in the Lao PDR, and as such it is an important element of food security [13]. Rice cultivation occupies more than 80% of the agriculture area and provides almost 80% of the inhabitants calorie in-take [14]. Interestingly, this country is located close to the possible center of rice domestication [15] and it is also considered one of the potential centres of origin of the most important rice fungal pathogen, *Magnaporthe grisea* [16]. These data make the investigation in this area particularly intriguing. Even though Bakanae disease (in Laotian language “Phanyad khaophu”) is present in the Lao PDR, it has not been considered economically important, as relatively low-input, family farming production systems prevail still in most rice-growing areas of the country [17]. Recently, disease incidence increased due to changes in agricultural practices, especially because of the introduction of higher-yielding but more susceptible rice varieties [18]. Although the presence of Bakanae disease in the Lao PDR has been observed since 1993 [14], the first formal report of *F*. *fujikuroi*, in the Lao PDR, was published in 2017 [19], and the etiology of the disease has been clearly defined. *Fusarium moniliforme* (*nomen confusem*) was reported previously as the causal agent of the disease, but cultures were not deposited in an internationally recognized culture collection [1]. *Fusarium moniliforme* was recognized as a *nomen confusem* when the *Gibberella fujikuroi* species complex was the subject of intensive taxonomic studies, and mating populations (MP) and other criteria were used to distinguish species belonging to this complex [20, 21]. *Fusarium fujikuroi* belongs to mating population C (MP-C), classified on its respective ability to exchange genetic material through sexual recombination [22]. However, the separation between *F*. *fujikuroi* and *F*. *proliferatum* is not so clear. Indeed, hybrids between these two species have been reported [23]-.

Elucidating the population structure of a phytopathogenic fungus allows the evaluation of its evolutionary potential; this is an important pre-requisite for a successful breeding program for disease resistance, for the management of resistant genes in the field and also for reliable risk evaluation of overcoming the action of selected fungicides [24]. Relatively few studies have been done on the genetic structure of *F*. *fujikuroi*: in California [25], Philippines [26, 27], Taiwan [28] and Italy [11].

Microsatellites (SSRs) are informative and reliable markers in population genetics due to their high discrimination power and reproducibility and have been widely used for genetic studies of plant pathogens [29, 30]. Recently, *F*. *fujikuroi* specific SSR markers have been designed and utilized for informative population genetic studies on Italian and Taiwanese collections of the fungus [11, 28].

This study pursued two main aims. The first was to investigate the genetic variability of *F*. *fujikuroi* in Lao PDR, i.e. in a low-input traditional largely rain-fed farming system by using SSR markers that were specifically developed for this species [11]. In order to gain a deeper insight, SSR analysis was integrated with a survey of mating type (MAT) distribution, and with pathogenicity and fungicide sensitivity tests (e.g. [25, 11, 28]). The second aim was to compare the results obtained in Lao PDR (i.e. in a low input traditional rain-fed system) with those obtained in Italy (i.e., in a high input modern irrigated system) using the same set of SSR markers. This comparison would enable an insight into the role of human activities in shaping the population structure of the fungus and hence its evolutionary potential.

## Materials and methods

### Sampling and isolation

**Fig 1:**
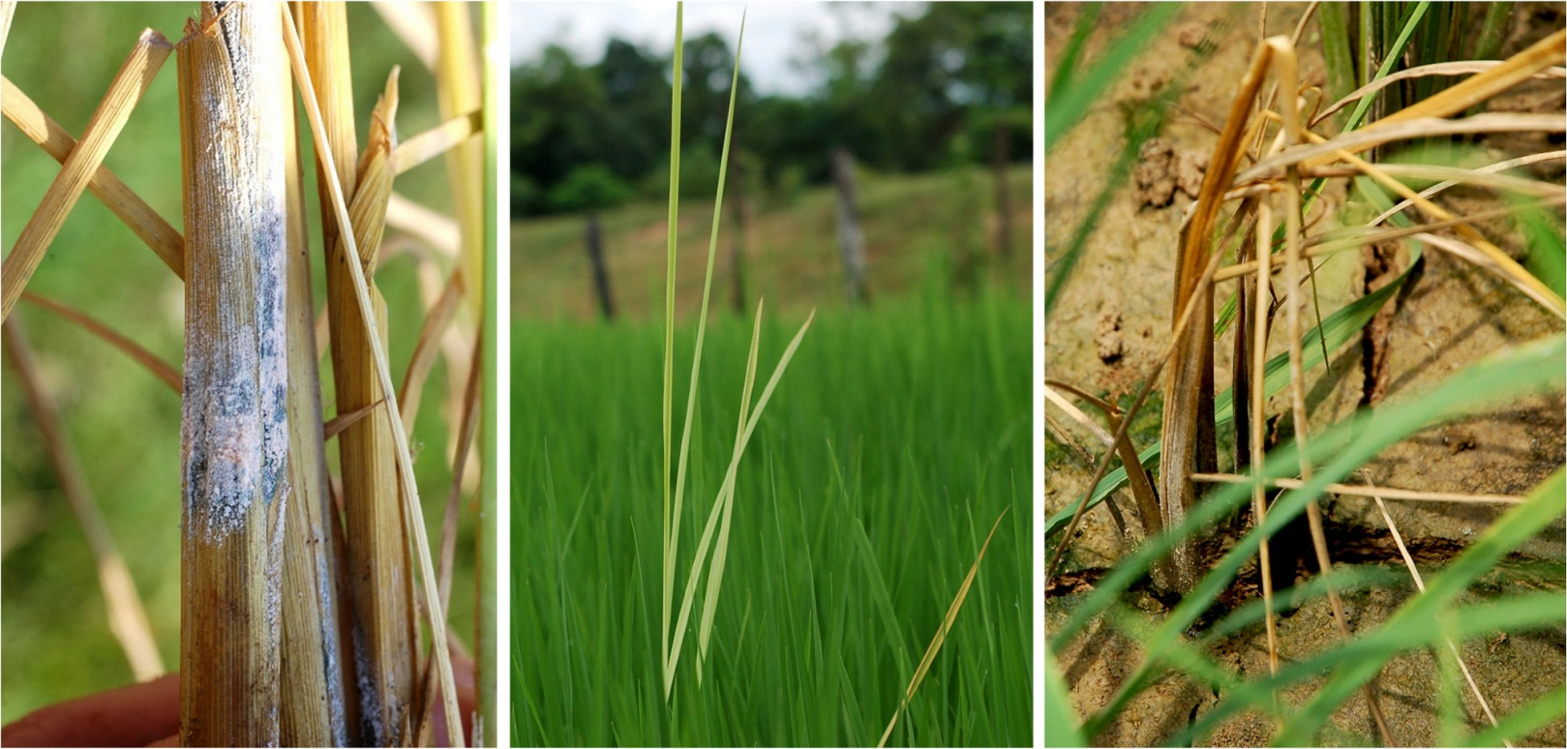
Symptoms caused by *Fusarium fujikuroi* on rice. Left to right: mycelium and sporodochia of *F*. *fujikuroi* on rice stem; plant with typical Bakanae symptom; plant with crown rot symptoms.

The present field study did not involve endangered or protected species and the sampling of the *F*. *fujkuroi* isolates characterized in the present work was carried out on private land after the owners gave permission to conduct the study on the sites. In August 2014, plants showing symptoms of plant elongation were collected from ten different rice fields (**Table 1**). The ten populations were from three geographical areas, one in Salavan province and two in Champasak province (**Table 1**). Each area was represented by two to five populations. Twenty to 25 plants with Bakanae symptoms (**Fig 1**) were sampled from each rice field. Isolates obtained from plants from the same rice field were considered a single population.

**Table 1:** Sampling locations in Laos of the ten populations of *Fusarium fujikuroi* analysed.

Population	Village	District	Province	GPS Coordinates
***Area 1***				
Pop 1	Dinkone	Kong	Salavan	15°38’32.8”N; 105°49’34.5”E
Pop 2	Kongkum	Kong	Salavan	15°35’51.5”N; 105°48’24.3”E
Pop 3	Nongkhulu	Kong	Salavan	15°31’04.3”N; 105°46’26.1”E
***Area 2***				
Pop 4	Sonefak	Sanasomboune	Champasak	15°17’54.2”N; 105°44’16.5”E
Pop 5	Sivilai	Sanasomboune	Champasak	15°13’49.4”N; 105°43’49.4”E
Pop 6	Sivilai	Sanasomboune	Champasak	15°12’57.9”N; 105°44’04.2”E
Pop 7	Donkhor	Pakse	Champasak	15°09’47.7”N; 105°45’02.2”E
Pop 8	---	Pakse	Champasak	15°06’03.94”N; 105°46’11.42”E
***Area 3***				
Pop 9	Don Kong	(Island)	Champasak	14°07’22.47”N; 105°51’14.47”E
Pop 10	Don Kong	(Island)	Champasak	14° 07'10.94"N; 105°49'14.15"E

Populations are sorted from North to South and in accordance with the geographical area of provenance.

The two rice cultivars from Don Khong (Island) were assumed to be traditional varieties.

Crown rot symptoms (**Fig 1**) were observed only within Pop 1 and Pop 2; in this case, isolations were made from plants with severe crown rot and dead plants.

Each plant was washed under running tap water and one basal stem section, 3 cm long, including the second node, was removed for isolation of the fungus. Each section was surface-sterilized for two min in sodium hypochlorite (2% [v/v]) and then rinsed twice with sterile water. Sections were dried on sterile paper under a laminar flow hood. Using sterile lancets, a small segment was removed from each stem section and plated on potato dextrose agar (PDA, SIGMA Aldrich) amended with Streptomycin sulphate 100 mg/L and Tetracycline 100 mg/L. The plates were incubated at 25 C under a 12-hour artificial light photoperiod for 4 to 6 d. All fungal colonies that developed were transferred onto PDA plates and used to produce single spore cultures [31].

### Identification of the isolates

#### Morphological identification

For morphological identification, isolates were grown on SNA (Spezieller Nährstoffarmer Agar), CLA (Carnation Leaf Agar) and PDA for 14 d at 25°C and 12-hr alternating artificial light and dark. Morphological identification of the isolated *F*. *fujikuroi* strains was performed following the specifications described in various manuals [32, 31, 23]. Macroconidia of *F*. *fujikuroi* were long, slender, almost straight and thin-walled. Microconidia were oval, obovoid with a truncated base produced in chains and in false heads from the conidiogenous cells, monophialides and polyphialides. Morphologically *F*. *fujikuroi* is almost indistinguishable from *F*. *proliferatum* but slight differences are observed: e.g. *F*. *fujikuroi* usually develops less polyphialides, its microconidial chains are shorter, and it produces more abundant sporodochia on agar media. Overall, 195 monoconidial isolates were obtained from the field samples. All isolates were stored at -80°C in the collection of the Department of Agricultural Sciences, University of Sassari, Italy.

#### Molecular identification

DNA was extracted from *F*. *fujikuroi* cultures grown on PDA at 25°C for 7 d following the protocol described by Aljanabi and Martinez [33]. Briefly, 100–150 mg of fresh mycelium was homogenized in 400μL lysis-buffer (10mM Tris-HCl, 2mM EDTA, 0.4M NaCl; 20% SDS, 28μL proteinase K (20 μg ml^-1^) and incubated at 60°C for 60 min. Following centrifugation, 300μL of a saturated NaCl solution was added to the supernatant and a second centrifugation step was carried out. DNA was precipitated with ice-cold isopropanol. To obtain high-quality DNA templates, samples were purified using the NucleoSpin^®^ Gel and PCR Clean-up (Macherey-Nagel, Düren, Germany) following the manufacturer’s instructions.

Morphological identification was confirmed with a species-specific PCR using primer pairs *Fuji1F* (5’-ACGTGTCAAACTAAACATTCGA-3) and *TEF1R* (5’GCGACAACATACCAATGACG-3) resulting in a 179 bp band for *F*. *fujikuroi* [34]. Briefly, in 20 μl of PCR reaction, 10× *Taq* amplification buffer, 0.5mM of dNTPs; 1.5mM of MgCl2; 0.4 μM of each primer and 1 U *Taq* DNA polymerase (Invitrogen) were mixed and submitted to amplification: a first cycle of 5 min at 94°C, followed by 35 cycles with denaturation at 94°C for 40 sec, 63°C for 40 sec, 72°C for 40 sec and a final extension at 72°C for 5 min. The visualization was performed in agarose gel (1.5%). Eleven isolates did not result in a PCR product. Their morphological re-analysis proved that they belonged to either *F*. *anthophilum* or the *F*. *incarnatum-equiseti* species complex—FIESC), and to the genera *Gliocladium* and *Arthrobotrytis*. Thus, 184 out of the initial isolates (94.3%) were confirmed as *F*. *fujikuroi* and subjected to the succeeding analysis.

### Genetic analyses

#### Mating-type analysis

The mating type of *F*. *fujikuroi* isolates was determined by PCR analysis as described by Valente et al. [11] using the two primer pairs described by Martin et al. [35]: M1C-5/M1C-13, specific for MAT-1 idiomorph, and M2C-9/ M2C-4 for MAT-2 idiomorph. Mating type analysis was carried out for 184 isolates of *F*. *fujikuroi*.

#### Microsatellite (SSR) analysis

The analysis was conducted using 17 SSR from the 19 SSR adopted by Valente et al. [11] to characterize an Italian collection of *F*. *fujikuroi*, excluding TUZ4 and TUZ18 (see Table 2 of ref. [11]). Among the 17 SSR markers, three were on chr1, three on chr2, four on chr3, two on chr4 and one on each of the chromosomes 5, 6, 7, 8 and 9. The physical distance among linked markers spanned from 76 kb to 586 kb on chr1 (average = 388 kb), from 571 kb to 3,389 kb on chr2 (average = 2,260 kb), from 191 kb to 1,388 kb on chr3 (average = 829 kb) and was of 1,090 kb on chr4.

SSR typing was successful for 175 isolates out of the 184 that were characterized for MAT type. Amplification of polymorphic SSR loci was carried out as described by Schuelke [36] and Valente et al. [11]. The use of three primers enabled contemporaneous (i) amplification and (ii) fluorescent labeling of the SSR fragments: a sequence-specific forward primer with M13 tail (CACGACGTTGTAAAACGAC) at its 5' end, a sequence-specific reverse primer and the universal M13 primer, fluorescent-labelled with 6-carboxy-fluorescein (FAM) or hexachloro-fluorescein (HEX) at its 5’ end. For further details regarding PCR amplification protocols see Valente et al. [11]. PCR fragment lengths were analyzed in capillary electrophoresis (3100 Avant Genetic Analyzer).

### Phenotypic analyses

#### Pathogenicity

Pathogenicity tests were conducted on a subset of 33 isolates. These were chosen based on the results of the genetic population structure analysis. Indeed, based on SSR markers and the clustering method of Structure software, two main groups of isolates were identified (named G1 and G2; see the Results section). Isolates for pathogenicity test were chosen to represent both these groups (16 and 17 isolates from G1 and G2, respectively; see the Results section). Pathogenicity tests were undertaken by inoculating seeds with 1 mL of conidial suspension at a concentration of 1×10^5^ spores ml^-1^. One ml of sterile water was used as a control. Conidial suspensions were prepared as described by Valente et al. [11]. A randomized block design with three replicates of ten seeds each was used.

The test was conducted in the greenhouse with temperatures between 15 and 30°C. Seeds of the susceptible rice cultivar “Carnaroli” were grown in special pots (ROOTRAINERS^™^, Haxnicks, UK,) containing sterile soil autoclaved at 121°C for 20 min.

After 25 d, plantlets that had emerged were counted and recorded, and then removed from the soil. The disease severity was evaluated using a scale from 0 to 4 as described by Zainudin et al. [37] and modified by Valente et al. [11]. The scale spans over 5 classes: 0 = no symptoms; 1 = normal growth but leaves beginning to show yellowish–green and small necrotic lesions localized at the crown level; 2 = abnormal growth, elongated, thin and yellowish-green leaves; seedlings stunted, necrotic lesions on main root and crown; 3 = abnormal growth, elongated stems, chlorotic, thin and brownish leaves; seedlings also shorter or taller than normal, reduced root system with necrotic lesions on secondary roots and on basal stem; 4 = dead plants before or after emergence. Infection severities were calculated using the McKinney index [38]. Disease severity was evaluated considering separately two variables: the ‘% of dead plants’ before or after emergence (i.e. the % of plants falling into the 4^th^ class of the above-mentioned severity scale) and the ‘plant height’ calculated as the average height across the plants falling into the severity classes from 0 to 3.

#### Fungicide sensitivity

Twenty-one Laotian isolates out of the 33 isolates subjected to pathogenicity test (10 from G1 and 11 from G2) were also analyzed for their sensitivity to the fungicide prochloraz at a concentration of 1 mg/L. The fungicide treatment was also applied to 16 Italian isolates studied by Valente et al. [11] to allow comparison between isolates from Laos and Italy. Fungicide was added to the PDA substrate just before pouring the plates to avoid its degradation by higher temperature. A plug of PDA with the mycelium of each isolate was placed in the center of the plates. Colony diameters were measured after 3 and 6 d of incubation at 25°C in the dark. The experiment was replicated three times. Growth inhibition percentages were calculated with the following formula: [(growth with the fungicide—growth of the control) / (growth of the control)] × 100.

### Statistical analyses

#### Genetic data

Geographical populations. The percentage of polymorphic loci, the number of alleles (n_a_) and the average diversity over loci using the unbiased Nei’s gene diversity (H_E_,) [39] were calculated using Arlequin software ver. 3.5 [40] for each of the ten populations. The genetic divergence among populations (F_ST_) [41] was estimated using the hierarchical analysis of molecular variance (AMOVA) [42]. Two AMOVA models were used. In the first model, the total SSR variance was partitioned into two levels: among populations and among individuals [within population]. This model answers the question of whether populations showed different allele frequencies (gene pools). In the second model, three levels were considered: among populations, among mating types [within populations] and among individuals [within mating types, within populations]. This model answers the question if, *on average*, opposite mating types within a population were differentiated, as expected if gene flow (recombination) is not having a role within populations. Moreover, for each population separately, AMOVA was applied considering the levels: between mating types and among individuals [within mating types]. This allowed us to investigate gene flow among types separately for each population. Finally, to estimate the global level of genetic differentiation between the two mating types after deducing the effect of population divergence, we applied a model with three levels: mating types, populations [within mating types], and isolates [within mating types, within populations]. AMOVA was conducted using Arlequin software [40]. Wright’s F-statistics [41] confidence intervals (C.I.) were calculated by bootstrapping over loci (1000 reps). Genetic distances among pairs of populations were determined using the F_ST_ statistics; their significance was tested using 10^5^ permutations.

Individual based analysis. To cluster isolates and detect genetic populations, Structure ver. 2.3.4 [43] was adopted. This uses a Bayesian approach with Markov chain Monte Carlo (MCMC) method to estimate allele frequencies in each cluster and assigns each individual to different clusters according to a membership coefficient (q_i_). The admixture model was run using the options ‘correlated allele frequencies among populations’ and ‘infer the degree of admixture (α) by the data’. For each K (number of assumed populations), 20 runs (burn-in length, 100 000; iterations, 200 000) were carried out, and the most likely number of K was determined using the ΔK statistic [44], as implemented in Structure Harvester [45]. The evolutionary relationships of the 175 *F*. *fujikuroi* isolates were further investigated obtaining a neighbour-joining tree based on the pairwise differences between isolates with MEGA X [46].

We used the function *snapclust* [47] in the R package *adegenet v2*.*1*.*1* [48] to complement Structure analysis. This enabled a fast likelihood optimization method combining both model‐based and geometric clustering approaches, which uses the Expectation‐Maximization (EM) algorithm to assign genotypes to populations. We looked for the optimal number of clusters using *snapclust*.*choose*.*k* (allowing a maximum of 20 populations) and we chose the model with the minimum BIC (Bayes Information Criterion). Initial group memberships for *snapclust were* chosen using the k‐means algorithm (pop.ini = “kmeans” and with a maximum number of iterations, *max*.*iter* = 100). The analysis successfully converged at the second iteration. To describe the clusters identified by *snapclust*, a discriminant analysis of principal components (DAPC) [49] was performed considering the *snapclust* clusters as *a priori* groups for DAPC [47]. DAPC attempts to summarize genetic differentiation between groups (those identified by *snapclust* in this context) and does not assume a population genetics model. Instead, it transforms the data using PCA and then performs discriminant analysis on the number of principal components retained. The number of retained principal components was determined using the cross-validation function *xvalDapc* and based on the lowest root mean square error (30 replicates). DAPC was performed with the R package *adegenet v2*.*1*.*1* [48].

Spatial analysis. To test the association between genetic and spatial distance among isolates the Mantel test was performed [50]. A spatially explicit multivariate method, the spatial analysis of principal components (sPCA), was also adopted [51]. The sPCA method imposes no genetic assumption based on the mating system, population structure or alleles frequency model. The method allows finding independent synthetic variables (spatial principal components, sPCs) that capture either genetic diversity and spatial trends. Furthermore, sPCs seek to maximize the product between genetic variance and spatial autocorrelation in allele frequencies [52]. Spatial autocorrelation is quantified by the Moran I Index [52]. This is expected equal to zero when there is random spatial distribution of the genetic variation. When Moran I is positive (with the highest possible positive value being 1) allele frequencies observed at neighbouring sites tend to be similar while frequencies at distant sites tend to be dissimilar. This scenario is referred to as positive spatial autocorrelation or “global structure” and reflects the presence of large patches or clines. When Moran index is negative (with the maximum possible values being -1) allele frequencies tend to be dissimilar at a short spatial scale giving rise to negative spatial autocorrelation also referred to as “local structures” [51]. To perform sPCA the connection network between pairs of isolates was built using the inverse Euclidean distance as spatial weights. As suggested in Jombart and Collins [53], we applied non-parameteric randomized Monte Carlo tests (referred to as “global” and “local” tests) to determine the statistical significance of the inferred structures. All these analyses were conducted in R using the library *adegenet* [48]. Spatial autocorrelation analysis was performed using GenAlex ver 6.5 [54]. Correlograms were obtained by averaging the autocorrelation coefficient (r) within each distance class. Following Banks and Peakall [55] we declared the significance of the heterogeneity tests [56] at the 1% cut-off.

Multilocus analysis. Multilocus linkage disequilibrium (LD) was quantified using the r_d_ index, a relative measure of panmixis, using MultiLocus ver. 1.2 software and tested with 1000 randomizations [57]. The percentage of SSR pairs in LD was determined with Arlequin software adopting 10^5^ randomizations. To assess the effect of clonality due to possible epidemic structure on LD estimates, we compared the results of two analyses: one using all individuals and one in which each distinct multilocus genotype was represented only once (e.g. [58, 59, 60]). We verified that repeated haplotypes were ‘real clones’ by calculating P_sex_, the probability that the same genotype arose in several individuals within a population by independent reproduction events [61, 62] using MLGsim2.0 [63]. The program performs simulations of populations under random mating to assess the significance of P_*sex*_ values. Identical haplotypes with significantly low P_sex_ values (<0.05) may be considered as belonging to the same clonal lineage.

Mating type distribution. The null hypothesis of the 1:1 ratio of the two mating types of the fungus, both at the regional level and within each field, was evaluated using the χ2 test (e.g., [64, 65]). The χ^2^ values are measures of departure from the 1:1 expectation [66]. In addition, an exact binomial test for goodness of-fit was performed. When sample sizes are small, the exact binomial test is more accurate than the χ^2^ test [67, 65, 64]. The differences among populations for mating-type frequencies were evaluated by calculating the F_ST_ with Arlequin software.

#### Phenotypic data

Differences between isolates for pathogenicity as measured by the McKinney Index, or % of dead plants, or plant height were evaluated with an ANOVA model comprising isolates and replicates. Differences between inoculated plants and the control were assayed by Dunnet test.

Differences in fungicide sensitivity (as mycelial growth inhibition with 1 mg/L of prochloraz at three and six days) were tested with an ANOVA model including isolates, time, isolates × time and replicates. Differences between groups (or genetic clusters) of isolates were tested using ANOVA and fitting a model with groups (or genetic clusters) and replicates as variables. Mean separation was obtained by the Tukey-Kramer multiple comparison test. The association between nominal variables was evaluated by the χ^2^ test, while association between continuous variables was quantified by parametric Pearson's r coefficient. All analyses were conducted with the SAS Institute software package JMP version 10.

## Results

### Diversity, linkage disequilibrium

Gene diversity (H_E_) for each locus are reported in **S1 Table**, while diversity estimates for the 10 Laotian populations of *Fusarium fujikuroi* are presented in **Table 2**. Populations from different areas had different levels of diversity as measured by the % of polymorphic loci and gene diversity (H_E_) (Kruskal-Wallis test: P = 0.0186 and P = 0.044, respectively). Populations of Area 1 were more diverse than those of Area 2 (Wilcoxon test: P<0.05) with Area 3 in an intermediate position (**Table 2**). Differences among areas based on the number of alleles were not significant (Kruskal-Wallis test: P = 0.054).

**Table 2:** Diversity estimates for the 10 Laotian populations of *Fusarium fujikuroi*.

Population	N. isolates	Pol. loci (%)^a^	N_a_	^a^^,^^b^Average H_E_
***Area 1***				
Pop 1	23	17 (100)	3.94	0.57
Pop 2	24	17 (100)	4.12	0.56
Pop 3	16	16 (94.1)	2.94	0.49
*Average*	21	16.7 a	3.67	0.54 a
*Total*	63	17	5.35	0.58
***Area 2***				
Pop 4	14	14 (82.4)	2.59	0.42
Pop 5	15	14 (82.4)	2.59	0.45
Pop 6	11	10 (58.8)	1.71	0.22
Pop 7	18	14 (82.4)	2.29	0.19
Pop 8	16	14 (82.4)	2.47	0.32
*Average*	14.8	13.2 b	2.33	0.32 b
*Total*	74	14	3.53	0.42
***Area 3***				
Pop 9	19	15 (88.2)	2.71	0.45
Pop 10	19	16 (94.1)	2.77	0.36
*Average*	19	15.5 ab	2.47	0.41 ab
*Total*	38	16	3.18	0.50
*Grand Mean*	17.5	14.7 (86.5)	2.81	0.40
*Grand Total*	175	17 (100)	5.94	0.53

Populations are listed from North to South and according to their area of origin. For each statistic, values for each area and for the total sample are also provided.

^a^ for the columns pol. loci (%) and average H_E_ the average values of areas not connected by same letter are significantly different at Wilcoxon test (P<0.05)

^b^considering all loci

Within the sample of 175 isolates, 97 SSR multilocus profiles (haplotypes) were identified (**Table 3**).

**Table 3:** Multilocus statistics for the 10 Laotian populations of *Fusarium fujikuroi* by area of origin.

Population	N. isolates	Distribution of haplotype frequencies										N. haplotypes	Ratio Iso./Hap	Haplotype diversity	Multilocus LD (r_d_)	
		1	2	3	4	5	6	7…	10	11…	16		.		All isolates	Clone corrected
**Area 1**																
Pop1	23	16	2	1								19	1.2	0.98	0.12***	0.09***
Pop 2	24	18	3									21	1.1	0.99	0.08***	0.07***
Pop 3	16	8	2		1							11	1.5	0.93	0.35***	0.27***
Mean	21.0	14.0	2.3	1.0	1.0							17.0	1.3	0.97	0.18	0.14
**Area 2**																
Pop 4	14	5	2				1					8	2	0.81	0.34***	0.13***
Pop 5	15	7		1		1						9	1.7	0.88	0.47***	0.34***
Pop 6	11	5		2								7	1.6	0.89	0.21***	0.19***
Pop 7	18	3	1				1	1				6	3	0.76	0.64***	0.56***
Pop 8	16	5	1	1			1					8	2	0.84	0.32***	0.20***
Mean	15.0	4.3	1.0	1.5			1.0	1.0				7.0	2.2	0.83	0.40	0.28
**Area 3**																
Pop 9	19	15			1							16	1.2	0.96	0.28***	0.23***
Pop 10	19	7	1						1			9	2.1	0.73	0.37***	0.13***
Mean	19.0	11.0	1.0		1.0				1.0			12.5	1.7	0.85	0.33	0.18
Grand Mean	17.5											11.3	1.7	0.88	0.32	0.22
Total	175	70	13	4	3	3	1	1		1	1	97	1.8	0.81	0.13***	0.09***

r_d_ = index of multilocus linkage disequilibrium (LD) (57); this was calculated both considering all isolates and clone-corrected samples (***P<0.001).

Among these 97 haplotypes, 70 were unique, i.e. each of them identified a specific isolate and 27 (that accounted for 105 isolates) were repeated from a minimum of two times up to 16 times (**Table 3**). Among the 27 repeated haplotypes, thirteen (~50%) were “private” (i.e., were repeated only within population).

All populations contained repeated haplotypes (**Table 3**), i.e. different isolates that shared the same genetic profile. The ratio isolates/haplotypes ranged between 1.1 (Pop 2) and 3.0 (Pop 7). It tended to be lower for populations from Area 1 compared with populations from Area 2, albeit differences among areas were not significant (Wilcoxon non-parametric test, P = 0.057).

The multilocus index of linkage disequilibrium (r_d_) was highly significant for all populations (P<0.001 in all cases; **Table 3**). If clone-corrected samples were considered, r_d_ value was reduced in all populations, but remained highly significant (P<0.001 in all cases) despite the reduction of sample size, i.e. of the power of the test (**Table 3**). After eliminating loci in close linkage (at a distance below <200 kb), overall, the changes of r_d_ values were small (**S2 Table)**. Depending on the population, the r_d_ slightly increased or decreased and, in all cases, P values remained <0.001. This was true either considering all isolates or clone-corrected samples. Moreover, we also evaluated the % loci pairs that were in LD within populations, distinguishing between linked and unlinked SSR loci (**S3 Table**). The average % of loci in LD within populations was 54.3%, with 47.4% for linked loci *versus* 54.9% for unlinked loci. The % of pairs in LD for linked and unlinked loci was similar across all populations except one population (Pop 1) where the LD between unlinked loci was higher than for linked loci.

Based on MLGsim analysis, most repeated haplotypes were confirmed as “true” clones (**Table 4**).

**Table 4:** Distribution of repeated haplotypes.

		Area 1			Area 2					Area 3	
		1	2	3	4	5	6	7	8	9	10
Haplotype	Isolates	(23)	(24)	(16)	(14)	(15)	(11)	(18)	(16)	(19)	(19)
I	5	**3**^**+**^	0	**2**^**+**^	0	0	0	0	0	0	0
II	5	1	0	**4**^**+**^	0	0	0	0	0	0	0
III	3	1	0	**2**^**+**^	0	0	0	0	0	0	0
IV	2	**2**^**+**^	0	0	0	0	0	0	0	0	0
V	2	**2**^**+**^	0	0	0	0	0	0	0	0	0
VI	2	1	0	1	0	0	0	0	0	0	0
VII	2	1	0	1	0	0	0	0	0	0	0
VIII	3	0	1	0	0	1	0	0	0	1	0
IX	2	0	**2**^**+**^	0	0	0	0	0	0	0	0
X	2	0	**2**^**+**^	0	0	0	0	0	0	0	0
XI	2	0	**2**^**+**^	0	0	0	0	0	0	0	0
XII	2	0	0	1	1	0	0	0	0	0	0
XIII	11	0	0	0	**6**^**+**^	0	3	2	0	0	0
XIV	2	0	0	0	**2**^**+**^	0	0	0	0	0	0
XV	2	0	0	0	**2**^**+**^	0	0	0	0	0	0
XVI	2	0	0	0	1	0	1	0	0	0	0
XVII	5	0	0	0	0	**5**^**+**^	0	0	0	0	0
XVIII	3	0	0	0	0	**3**^**+**^	0	0	0	0	0
XIX	3	0	0	0	0	0	3	0	0	0	0
XX	7	0	0	0	0	0	0	**7**^**-**^	0	0	0
XXI	6	0	0	0	0	0	0	6	0	0	0
XXII	16	0	0	0	0	0	0	0	**6**^**+**^	0	**10**^**+**^
XXIII	4	0	0	0	0	0	0	0	**3**^**+**^	0	1
XXIV	4	0	0	0	0	0	0	0	**2**^**-**^	0	**2**^**+**^
XXV	4	0	0	0	0	0	0	0	0	**4**^**+**^	0
XXVI	3	0	0	0	0	0	0	0	1	1	1
XXVII	2	0	0	0	0	0	0	0	0	1	1

The 10 populations are listed by area and sorted from North to South. The number of isolates analysed in each population is between parentheses. In bold: significant clonal lineages (“true clones”) when MLGsim analysis was performed for each population separately

+ = P<0.001,— = P<0.05.

Black contour line: significant (P<0.001) clonal lineages when MLGsim analysis was performed considering all 175 isolates as a unique population.

The probability that repeated haplotypes within a population originated from distinct reproductive events was low overall. Indeed, when populations were considered separately, P_sex_ was low (from 5.12×10^−3^ to 3.3×10^−11^) and significant in 21 out of 25 (84.0%) cases (P <0.001 in 19 cases and P<0.05 in two cases) (**Table 4**). All populations contained at least one “true” clone except for Pop 6. In this case, for three isolates bearing haplotype XIII (P_sex_ = 0.015) and the three isolates bearing haplotype IXX (P_sex_ = 0.002) significance was not reached (P = 0.232 and P = 0.090, respectively) (**Table 4**). Thus, the hypothesis that they resulted from sexual reproduction could not be discarded. The same applied for haplotypes XIII and XXI of Pop 7 (P_sex_ = 0.027, P = 0.214; P_sex_ = 0.012, P = 0.150; respectively). Moreover, when MLGsim analysis was repeated considering all 175 isolates as a unique population, all repeated haplotype resulted in “true” clones (P_sex_ from 2.61×10^−4^ to 0; P<0.001 in all cases) except for haplotype XII (P_sex_ = 2.61×10^−4^, P = 0.183; **Table 4**). “True” clones were more often shared among two populations of the same area. However, populations quite far apart (>100 km) can share true clones, e.g., haplotype VIII that was in common between populations 2, 5 and 9 (**Table 4**).

### Population structure

#### Geographical (field) populations

The genetic divergence among areas was low (F_CT_ = 0.042) but significant (P = 0.021; **Table 5**).

**Table 5:** Results of global AMOVA (as a weighted average over loci) to test for geographical structuring.

Source of variation	F	^1^P	^2^I.C. 95%	^2^I.C.99.9%
*All isolates*				
Areas	0.042 (F_CT_)	0.021	0.004--0.089	-0.013--0.125
Populations[areas]	0.203 (F_SC_)	<10^−5^	0.177--0.228	0.156--0.244
overall	0.237 (F_ST_)	<10^−5^	0.204--0.273	0.179--0.304
*Clone-corrected*				
Areas	0.015 (F_CT_)	0.079	-0.006--0.037	-0.016--0.053
Populations[areas]	0.079 (F_SC_)	<10^−5^	0.057--0.103	0.045--0.122
Overall	0.093 (F_ST_)	<10^−5^	0.071--0.116	0.056--0.133

^1^tested by permutation (over 10^6^ replicates)

^2^bootstrap percentile values (over 20000 bootstraps)

The average divergence among populations within areas was moderate and highly significant (F_SC_ = 0.203; P<10^−5^). This resulted in an F_ST_ among populations of 0.237 (P<10^−5^), i.e. 23.7% of the total SSR variation was allocated among populations. After clone-correction, F values were reduced. The divergence among areas became not significant (F_CT_ = 0.015; P = 0.071) while the average divergence of populations within areas (F_SC_ = 0.079) and the total population divergence (F_ST_ = 0.093) remained significant (P<10^−5^ in both cases).

**Fig 2:**
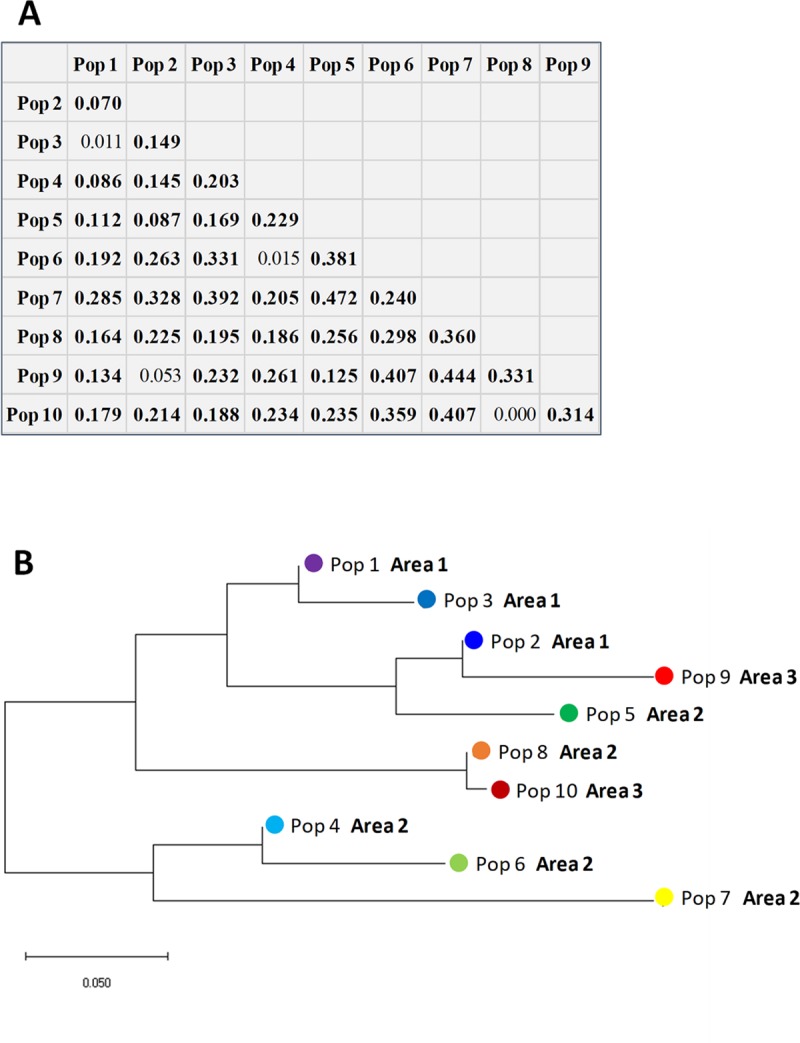
Relationships among the ten geographical populations of *Fusarium fujikuroi*. A) pairwise F_ST_ matrix based on AMOVA (without clone correction). B) Neighbour-joining tree. The optimal tree with the sum of branch length = 0.706 is shown. The tree is drawn to scale, with branch lengths in the same units as those of the evolutionary distances used to infer the phylogenetic tree (F_ST_). The analysis was conducted in MEGA X.

When pairs of populations were considered (**Fig 2A**) F_ST_ values ranged from 0.000 (no divergence) to 0.472 (strong divergence).

Among the 45 population pairs, four (8.9%; pairs 1–3, 2–9, 4–6 and 8–10) were not significantly (P>0.05) divergent. The dendrogram showing the relationships among the ten geographical populations of *F*. *fujikuroi*, evidenced that populations of Area 1 tended to be separate from those of Area 2 (**Fig 2B**).

After clone correction, the highest F_ST_ was 0.319 (**S1A Fig**). Pairwise F_ST_ obtained using all isolates or after clone-correction were strongly correlated (Mantel test: R^2^ = 0.52, P<10^−5^) consequently, the relationships among populations remained substantially unchanged (**S1B Fig**).

#### Individual based analyses

**Fig 3:**
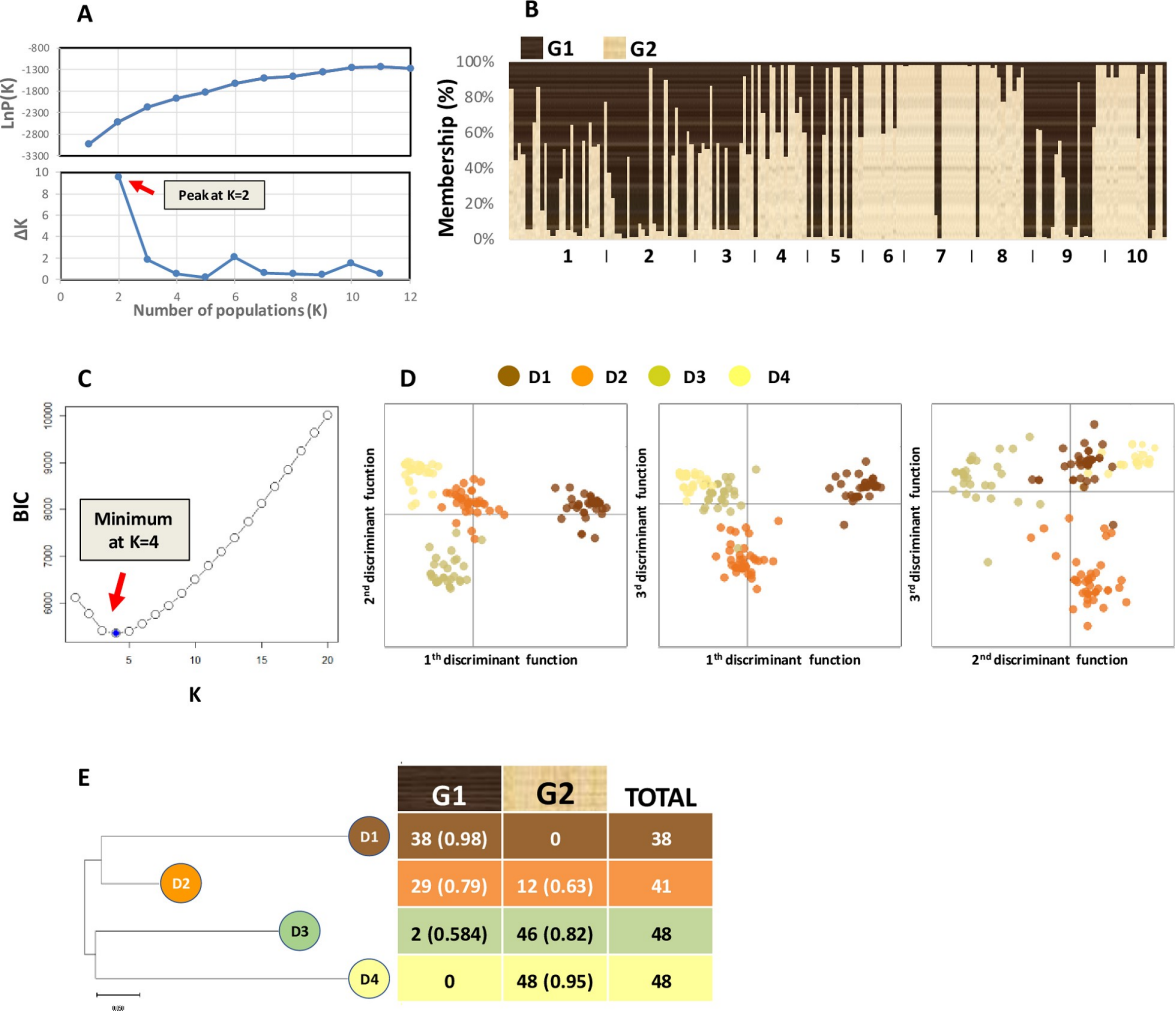
Results of the population structure analysis. A) Mean of the estimated of Ln of the probability of the data, LnP(K), calculated by Structure, as a function of the number of assumed populations, K. lower scatterplot: determination of the “true” number of populations based on the method of Evanno [44]. B) individual to groups assignment by Structure (K = 2). Individuals were sorted by population of origin and from North to South. The population numbers correspond to those specified in Table 1. C) Inferring the number of populations (K = 4) with *snapclust* based on the Bayesian Information Criterion (BIC). D) Results of Discriminant Analysis of Principal Component (DAPC) on the four clusters identified by *snapclust*. The three scatterplots reported the discrimination of the four clusters based on the first, second and third discriminant functions of DAPC analysis. E) Comparison between Structure and *snapclust*/DAPC groups. For each group, the number of isolates and the average q_i_ value returned by Structure for G1 or G2 are reported.

When all isolates were considered, Structure analysis indicated that the uppermost level of hierarchical structuring was at K = 2 (**Fig 3A and 3B**).

The two groups identified by Structure (named as G1 and G2) showed an intermediate level of genetic divergence (F_ST_ = 0.244) and comprised 69 (39.4%) and 106 (60.6%) (q_i_>0.5) of the isolates, respectively. Thirty-eight isolates were admixed between the two groups (0.30<q_i_<0.70). G1 showed higher diversity than G2 (H_E_: 0.54 vs 0.34). Multilocus LD (r_d_) was higher within G1 and G2 (r_d_ = 0.167 and r_d_ = 0.173, respectively) than within the group of admixed isolates (r_d_ = 0.140); however, it was significant in all cases (P<0.001). When the clone-corrected dataset (97 haplotypes) was subjected to Structure analysis, the existence of two main genetic groups was confirmed (**S2 Fig**). The genetic divergence between G1 and G2 became 0.201 with only one individual admixed (0.30<q_i_<0.70). The multilocus LD in these two clone-corrected groups lowered (r_d_ = 0.041 for G1 and r_d_ = 0.151 for G2) but remained highly significant (P<0.001).

Based on *snapclust*, the minimum BIC was reached when four clusters were assumed (**Fig 3C**). Cross-validation indicated that, to conduct DAPC, 20 PCs should be retained (**S3 Fig**) that conserve 90.7% of the total variance. DAPC classification based on these 20 PCs was consistent with clusters identified by *snapclust* as all individuals were assigned to the clusters of origin (**S4 Fig**) which were named D1, D2, D3 and D4 (**Fig 3D**; **S5 Fig**). The first discriminant function separated D1 from the other groups (**Fig 3D**). The second discriminant function separated D3 from D4 with D1 and D2 in an intermediate position. The third discriminant function separated D2 from the other groups.

Structure and *snapclust*/DAPC results were highly correlated (**Fig 3E**). Indeed, G1 mainly splits into D1 and D2 while G2 mainly into D3 and D4. In detail, G1 contained 100% and 71% of the isolates classified as D1 and D2, respectively, while G2 contained 100% and 96% of isolates classified as D4 and D3, respectively. The isolates that were misclassified between the two methods were admixed (q_i_<0.70) based on the Structure analysis (**Fig 3E**).

### Spatial analysis

**Fig 4:**
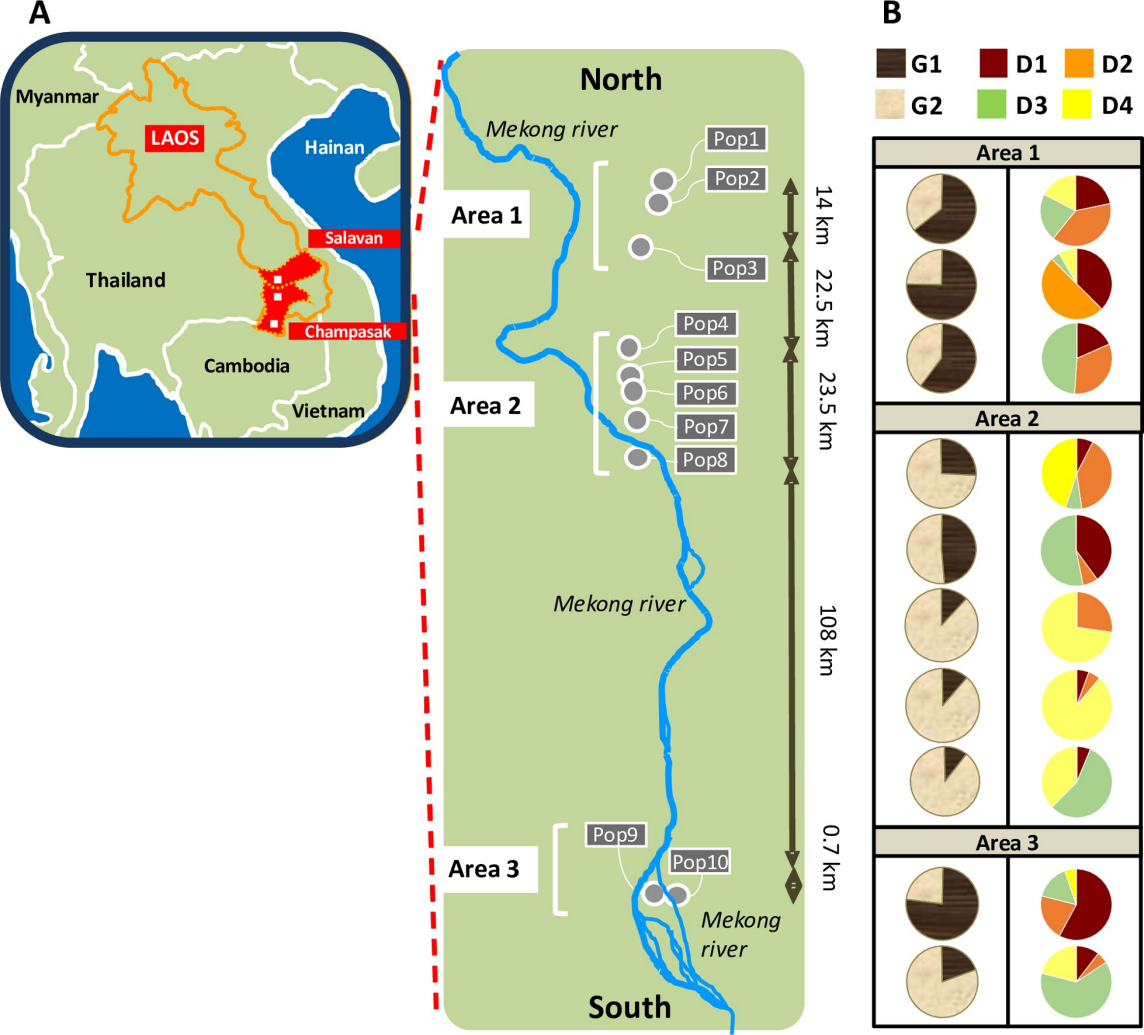
**Map of the 10 populations of *Fusarium fujikuroi* (A) and of the genetic groups detected by Structure and *snapclust*/DAPC analyses (B).** (A) The map is only for illustrative purpose and was drawn using Microsoft PowerPoint 2017. In the panel B, pies showed the average proportion of membership of the populations in each of the two genetic groups identified by Structure (G1 and G2; first column) and of the four groups identified by *snapclust/*DAPC (D1-D4, second column).

The Structure group G1 prevailed within the populations of Area 1 while G2 prevailed within those of Area 2 (**Fig 4**). In Area 3 both groups were equally represented. Among *snapclust*/DAPC groups, D2 was more frequent in Area 1 while D4 was more frequent in Area 2.

**Fig 5:**
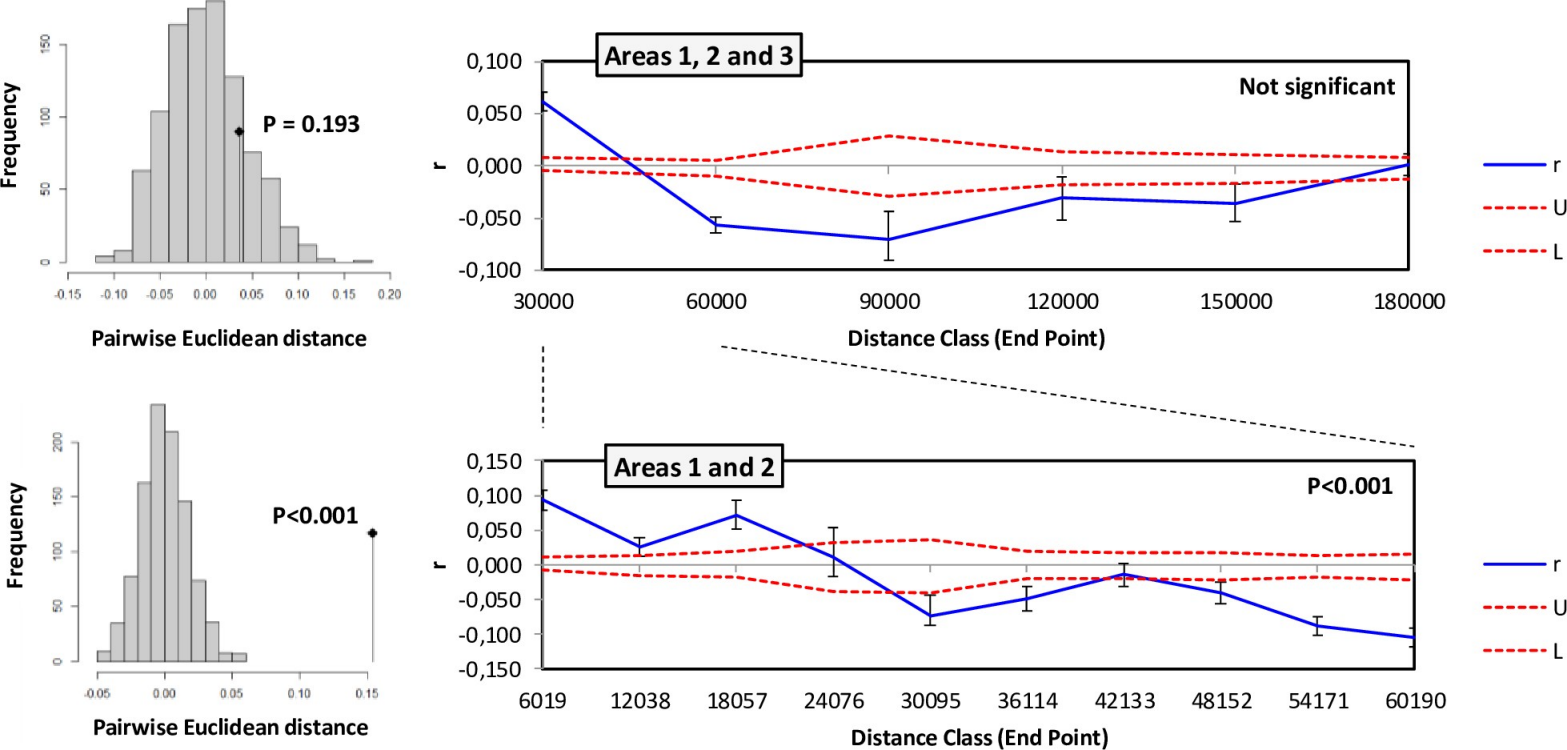
**Results of Mantel test of association between spatial and genetic distance (histograms on the left) and of Spatial autocorrelation analysis (panels on the right).** Top: results considering all 10 populations. Bottom: results considering only samples from Areas 1 and 2. Error bars bound the 95% confidence interval about r as determined by bootstrap resampling (1000 reps). Blue line: autocorrelation coefficient (r). Red dotted lines: upper (U) and lower (L) confidence limits bound the 95% confidence interval for the null hypothesis of no spatial structure for the combined data set as determined by permutation (1000 reps).

When all populations were considered, the Mantel test was not significant (P = 0.193) and the autocorrelogram was not significant (heterogeneity test: P>0.05; **Fig 5**).

However, when the analysis was restricted to Area 1 and Area 2, the Mantel test was highly significant (P<10^−5^) as also the autocorrelogram (heterogeneity test, P < 0.001; **Fig 5**) indicating that there was a gradient of genetic variation across the geographical range considered. Positive values of the autocorrelation coefficient (r) values (indicating isolates were more similar than expected compared to a random distribution) were found below ~20 km while negative values (isolates more dissimilar than expected) were found over ~50 km.

**Fig 6:**
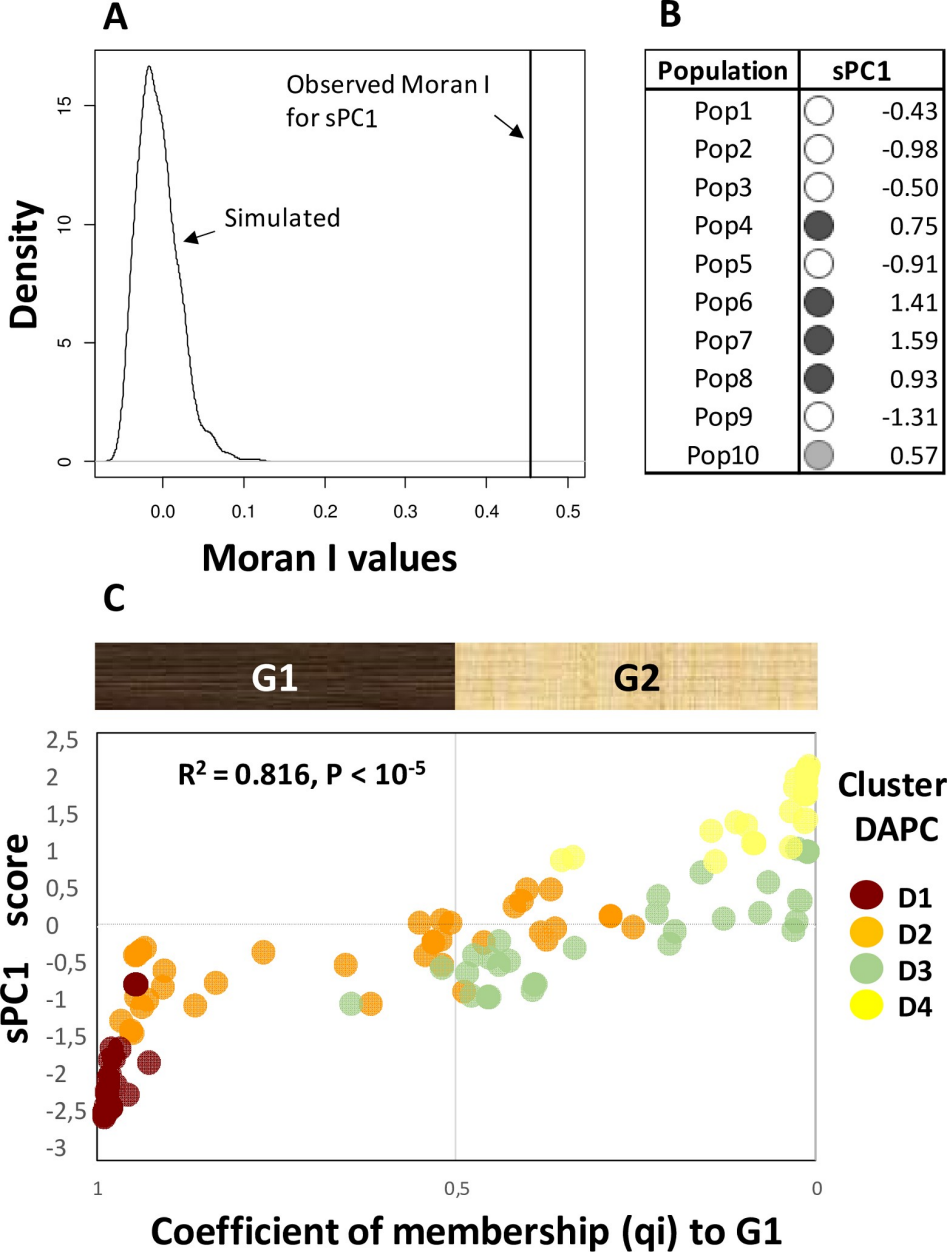
Results of spatial principal component analysis. A) Results of Monte Carlo simulation to test the significance of spatial autocorrelation for the first spatial principal component (sPC1). B) Average sPC1 scores for each of the ten populations of *F*. *fujikuroi* (populations are sorted from North to South). Blank, grey and black dots: strongly negative, intermediate and highly positive average scores for sPC1, respectively. C) sPC1 scores as a function of the coefficient of membership (q_i_) returned by Structure at K = 2. G1 and G2: the two groups identified by Structure. D1-D4: the four groups identified by *snapclust*/DAPC.

The bar plot of the eigenvalues of sPCA (**S6A and S6B Fig)** showed that the eigenvalues associated with positive spatial autocorrelation (that identify “global structures”, e.g. λ_1,_ λ_2,_ etc…) have values much higher than those associated with negative spatial autocorrelation (that identify “local structures”, e.g. λ_81,_ λ_80,_ etc…). This indicated that regional structure of allele frequency predominated over local structure. The bar plot of the eigenvalues of sPCA (**S6A and S6B Fig)** suggested that two sPCs (sPC1 and sPC2) with the first two positive eigenvalues (λ_1_ and λ_2_) identifying “global structures” should be retained. Differently, sPCs representing “local structure” should not be considered. Monte Carlo simulation corroborated this decision as indicated that the “global structure” was unlikely to arise from random spatial distribution of the sampled isolates (P<10^−5^; **S6C Fig**). The opposite was observed for the test for “local structure” (P = 0.162; **S6D Fig**). In more detail, sPC1 showed an eigenvalue (λ_1_) that was the first for the amount of genetic variance explained and the second for the strength of spatial autocorrelation (**S6B Fig**) with, however, a highly significant Moran index (I = 0.45; P<10^−5^) (**Fig 6A**).

sPC1 outlined the presence of a spatial structure associated with the North-to-South variation and particularly to the Areas 1 and 2 (**Fig 6B**). sPC1 was strongly correlated with the coefficient of membership (q_i_) returned by Structure at K = 2 (**Fig 6C**), i.e. sPC1 reflected the uppermost level of population structure. Thus, the identified positive spatial autocorrelation pattern was mainly due to the spatial arrangement of the Structure groups G1 and G2 as also of their associated *snapclust*/DAPC groups, D1-2 and D3-4, respectively (**Fig 6C**).

sPC2 showed an eigenvalue (λ_2_) that was the second for the variance explained and the first for spatial autocorrelation (**S6A and S6B Fig**) being Moran I = 0.48 (P<10^−5^; **S7 Fig**). This sPC highlights the genetic affinity among some of the northernmost populations and between the southernmost population of Area 2 and one population of Area 3 (**S7 Fig**). In the case of sPC2, the presence of positive spatial autocorrelation mainly depended on the distribution of the isolates attributed to the group D3 (**S7 Fig**).

### Mating type distribution

**Fig 7:**
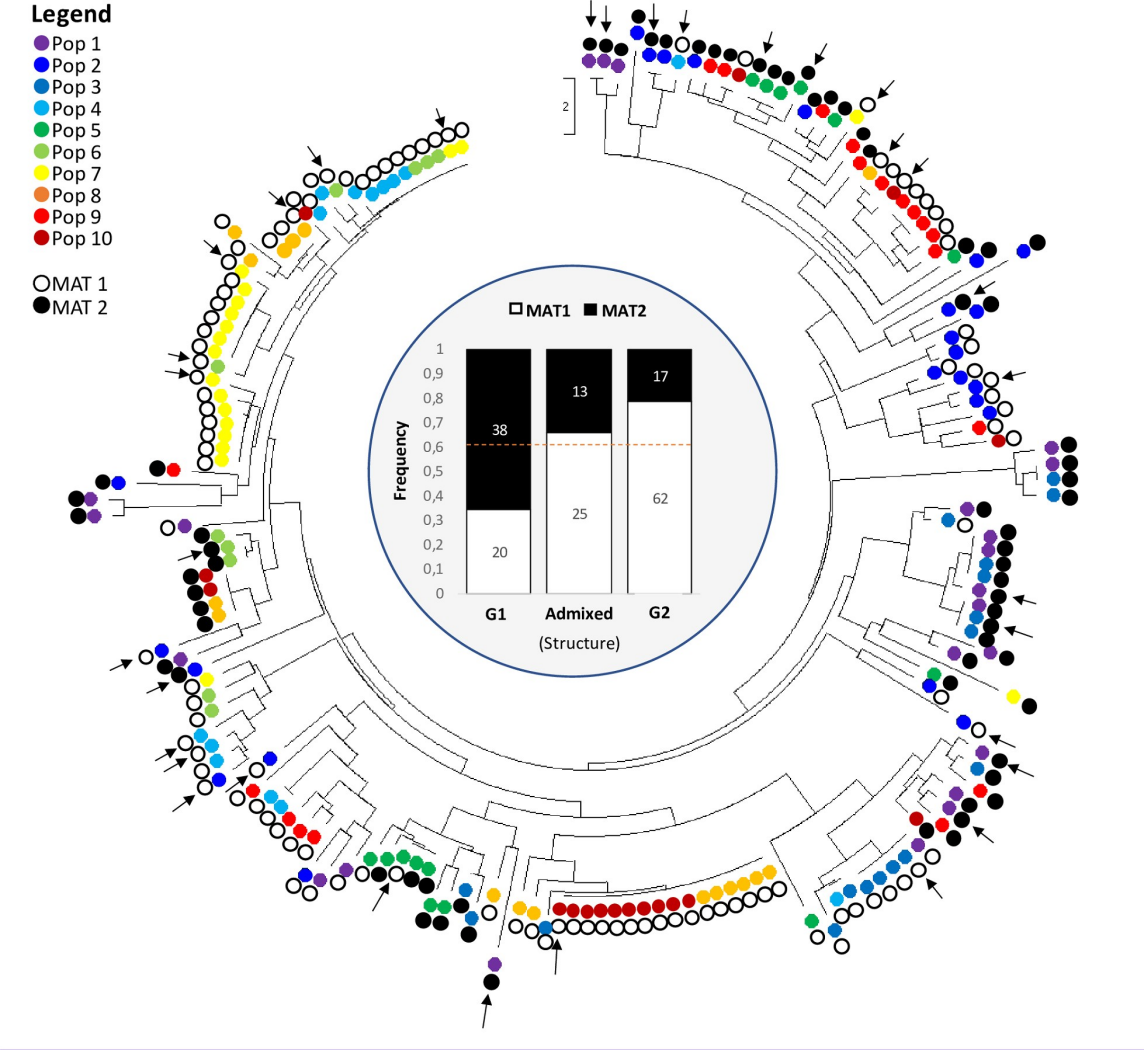
Evolutionary relationships of the 175 isolates of *Fusarium fujkuroi*. The evolutionary history was inferred using the Neighbour-Joining method implemented in MEGA X. The optimal tree with the sum of branch length = 199.74 is shown. The tree is drawn to scale, with branch lengths in the same units as those of the evolutionary distances used to infer the phylogenetic tree (number of differences between pairs of isolates). The internal and coloured dots represent the 10 geographical populations of the fungus. The external dots represent the two mating types: in white the MAT-1 and in black the MAT-2. The centre of the figure reports the frequencies of MAT idiomorphs within the genetic groups identified by Structure (G1 and G2) and the class of admixed isolates (0.30<q_i_<0.70). The dotted line represents the random expectation for mating type frequencies (61.1% of MAT-1 and 38.9% of MAT-2). The external black arrows indicate the 33 isolates subjected to pathogenicity test.

Isolates with the same MAT idiomorph frequently clustered together either when they came from the same populations or from geographically distant populations (**Fig 7**).

The two main SSR groups identified by Structure, G1 and G2, differed in MAT type frequencies (χ^2^ = 27.69, d.f. = 3, P<4.6×10^−6^). G1 showed an excess of MAT-2 types (χ^2^ = 17.35; d.f. = 1; P = 3×10^−5^) while G2 an excess of MAT-1 (χ^2^ = 10.00, d.f. = 1, P = 1.6×10^−3^) (**Fig 7**). The group of admixed individuals (0.30<q_i_<0.70) had a mating type ratio not skewed compared to the random expectation (χ^2^ = 0.35, d.f. = 1, P = 0.557). The analysis with the clone-corrected led to similar conclusions. The difference between G1 and G2 in mating type frequency was significant (χ^2^ = 15.96; d.f. = 2; P = 3.4×10^−4^) as G1 still exhibits an excess of MAT-2 (χ^2^ = 14.51, d.f. = 1; P = 1.4×10^−4^) while within G2, MAT frequencies were no longer skewed (χ^2^ = 1.44; d.f. = 1; P = 0.237).

Mating-type frequencies were unbalanced at all spatial scales considered (**Table 6**).

**Table 6:** Results of mating type frequency survey by area and population of origin.

	*MAT-1*: *MAT-2*		*Skew (All)*			*Gene flow*			
Origin	All	C.C.	χ^2^	P_χ2_	P_B_	F_ST (All)_	P _(All)_	F_ST (C.C)_	P _(C.C.)_
*Area 1*									
1	4:19	4:15	9.8	0.002	0.003	0.020	0.245	0.029	0.292
2	13:11	11:10	0.2	0.683	0.839	0.185	<10^−5^	0.192	<10^−5^
3	7:9	4:7	0.3	0.617	0.804	‘0.325	<10^−5^	0.149	0.124
Subtotal	24:39	19:32	3.6	0.059	0.077				
*Area 2*									
4	17:0	7:0	17.0	3.7x10^-5^	1.53x10^-5^	n.a.	n.a.	n.a.	-
5	5:12	1:7	2.9	0.090	0.143	0.123	0.031	n.a.	-
6	10:3	6:1	3.8	0.052	0.092	0.250	0.023	n.a.	-
7	17:1	5:1	14.2	1.6x10^-4^	1.5x10^-4^	n.a.	n.a.	n.a.	-
8	15:3	7:1	8.0	0.005	0.008	0.393	<10^−5^	n.a.	-
Subtotal	64:19	26:10	24.4	7.8x10^-7^	7.4x10^-7^				
*Area 3*									
9	11:8	7:8	0.5	0.491	0.648	0.091	0.039	0.157	0.040
10	16:3	7:2	8.9	0.003	0.004	0.255	<10^−5^	0.000	0.801
Subtotal	27:11	14:10	6.7	0.009	0.014				
**Total**	115:69	59:52	11.5	7.0x10^-4^	8.6x10^-4^	0.213^a^	<10^−5^	0.118^a^	0.006

Populations were from north to south. All = considering all isolates; c.c. = after clone correction; n.a. = not applicable because of the low polymorphism. P_χ2_, probability value for χ^2^ with 1 degree of freedom (d.f.); P_B_, likelihood of obtaining the observed result or a more discrepant result, given that the true ratio is 1:1. F_ST_ is calculated between the two mating types occurring within the same population.

^a^ The average genetic divergence between MAT types within populations was calculated as F_SC_ (based on the notation of Arlequin software) performing AMOVA with three hierarchical levels: among populations, between MAT types within populations and within MAT type within populations

When the entire sample of Laotian isolates was considered, MAT-1 was ~60% more frequent than MAT-2 (115:69; χ^2^ = 11.5, P = 7.0x10^-4^; **Table 6**). Areas differed in mating type frequencies (F_CT_ = 0.118) as MAT-2 tended to prevail within Area 1 (24:39; χ^2^ = 3.6, P = 0.059) while MAT-1 significantly prevailed within Area 2 (64:19; χ^2^ = 24.4, P = 7.8x10^-7^) and Area 3 (27:11; χ^2^ = 6.7, P = 0.009). Within areas, differences among populations were observed (F_SC_ = 0.204): for example, within Area 2 between Pop 4 (17:0) vs Pop 5 (5:12; **Table 6**). This translated in strong differences among populations in mating type frequencies (F_ST_ = 0.322).

The ratio MAT-1: MAT-2 deviated from 1:1 in five populations (Pop 1, 4, 7, 8 and 10; **Table 6**). Among these, only Pop 1 showed an excess of MAT-2 while MAT-1 prevailed in four populations, with Pop 4 being monomorphic for this idiomorph.

The average genetic divergence (F_SC_) between MAT types within-populations was 0.213 (P<10^−5^). When populations were considered separately, MAT-1 and MAT-2 were genetically divergent in seven populations with F_ST_ values ranging from 0.091 and 0.393 (P from 0.039 to 10^−5^; **Table 6**). In one population, Pop 1, divergence between MAT types was not significant (F_SC_ = 0.020, P = 0.245). In two cases (Pop 4 and Pop 7) divergence between MAT types (F_SC_) could not be computed because zero (Pop 4) or only one (Pop 7) isolate with MAT-2 were detected. It was of note that none of the populations had either MAT idiomorphs with balanced frequency and without genetic divergence.

After clone correction, the mating type frequencies were balanced in the overall collection (59:52). This was due to the reduction of MAT-1 frequency, i.e. MAT-1 showed more repeated genotypes than MAT-2. However, despite the reduction in sample size, the mating type remained unbalanced in Area 2 (26:10; χ^2^ = 11.5, P = 0.008). After clone correction, the average genetic divergence between mating types within populations was halved (0.118) but remained significant (P = 0.006; **Table 6**). Within Pop 2 and Pop 9, i.e. in the two cases of higher sample size (i.e., when the test had higher power), divergence was still significant (P<10^−5^ and P = 0.04, respectively).

### Pathogenicity

**Fig 8:**
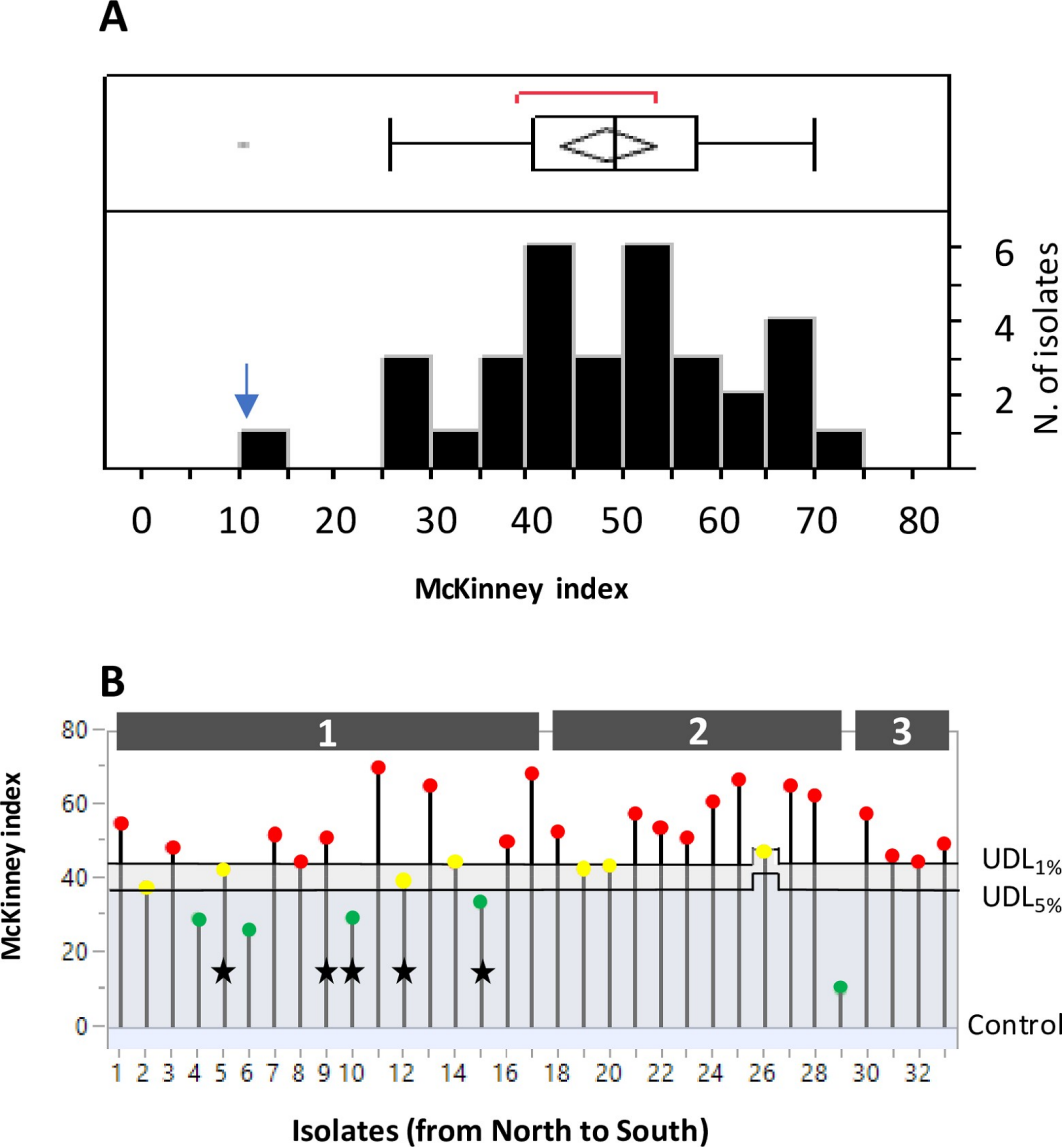
Results of pathogenicity test. **Variation in disease symptom severity (McKinney index) among 33 Laotian isolates of *F*. *fujikuroi*.** A) Frequency distribution; the blue arrow indicates the value for the control (uninfected plants). The ends of the boxes are the 25th and the 75th quantiles, the mid line identifies the median sample value, and the end of the whiskers are the outer-most data points. The red bracket outside of the box identifies the shortest half, which is the most dense 50% of the observations. B) Significance of the difference between each isolate and the control by Dunnet test. Red dots: isolates different from the control (P<0.05). Green: isolates not different from the control (P>0.05). UDL: upper Dunnet limit for α = 0.05 and α = 0.01. Black stars: isolates collected from dead plants showing stunting/foot rot symptoms. Isolates are sorted from North to South; grey numbered rectangles indicate the areas of origin of isolates.

Disease severity, measured by the McKinney index, was highly variable (**Fig 8**) and differences among isolates were highly significant (ANOVA: MS = 23853.38; F_33,65_ = 3.45, P < 10^−4^).

Among all isolates the McKinney index ranged from 10% to 70% with an average of 48.1% (**Fig 8**). However, five isolates did not differ statistically from the control (P > 0.05 at Dunnet test; **Fig 8**). Isolates with low virulence tended to be more frequent within Area 1 and less frequent when moving from North to South (**Fig 8**). The five isolates collected in Area 1 from dead plants showing stunting/foot rot symptoms were not significantly different from those collected from plants showing typical Bakanae symptoms (ANOVA; P > 0.05; **Fig 8**).

**Fig 9:**
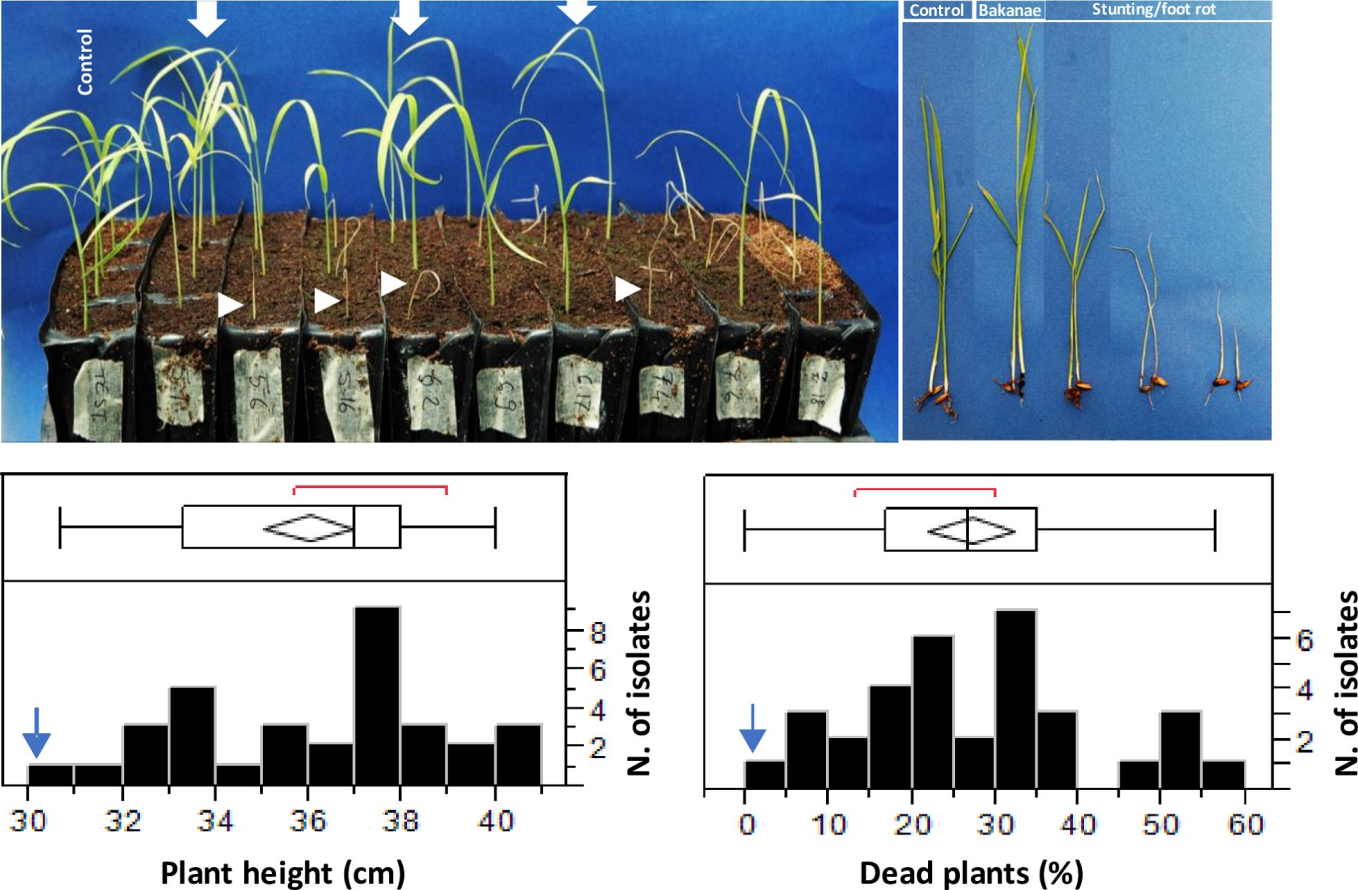
Results of pathogenicity test. **Variation for plant height and percentage (%) of dead plants.** Top picture on the left: control = uninfected plants; big white arrows = infected plants taller than the control (i.e., showing Bakanae symptom); small white arrows = dead plants or plants with stunting/foot rot symptoms. Top picture on the right: an example of the observed symptoms. Lower histograms: frequency distribution of plant height (as a measure of Bakanae symptom) and of the percentage (%) of dead plants. The ends of the boxes are the 25th and the 75th quantiles, the mid line identifies the median sample value, and the end of the whiskers are the outer-most data points. The red bracket outside of the box identifies the shortest half, which is the densest 50% of the observations.

Infected plants, on average, (excluding dead plants) had a height 20% higher than the control (36 cm vs 30 cm). However, the severity of symptoms was highly variable among the tested isolates (**Fig 9**).

Indeed, depending on the isolate, the height of infected plants was from 4.3% to 33.3% higher than the control (31.3 cm and 40.0 cm vs 30 cm, respectively; **Fig 9**). Furthermore, all isolates except one were able to induce plant death. Depending on the isolate, the percentage (%) of dead plants varied from 0% to 56.6% (control = 0.00%) and the mean was 26.7% (**Fig 9**). We did not observe significant correlation (P>0.05) between the ability to increase plant height and the ability to induce plant death.

### Mycelial growth

At three days, the average diameter of colonies was 3.0 cm, varying from 2.4 cm to 3.4 cm. After six days the average diameter was 5.5 cm, ranging from 4.4 cm to 6.5 cm. The diameter of the colony varied significantly among different isolates (**Table 7**).

**Table 7:** Results of analysis of variance to test difference in mycelial growth among 21 isolates of *Fusarium fujikuroi*.

Source of variation	D.f.	S.S.	F	P
Time	1	193.14	6109.71	<10^−4^
Isolate	20	13.95	22.07	<10^−4^
Isolate × time	20	3.84	6.08	<10^−4^
Replicates	2	0.13	2.13	0.13
Error	82	2.59		
Total	125	213.67		

D.f. = degree of freedom, S.S. = sum of squares, F = F ratio, P = significance level.

However, the interaction isolate × time was also significant indicating that growth rates varied in time differently across isolates. Indeed, some isolates had a constant growth rate while others started growing slowly and then accelerated their growth (**S8 Fig**). However, the correlation between colony diameters measured at three and six days was strong and highly significant (r = 0.662, n = 21; P = 0.0011), and the sorting of isolates was similar overall. There were no significant differences among areas, nor among isolates collected from plants with Bakanae or from dead plant showing stunting/foot rot symptoms (ANOVA, P > 0.05 in both cases). Moreover, there was no significant correlation (P>0.05) between colony growth rate and McKinney disease severity.

### Fungicide resistance

**Fig 10:**
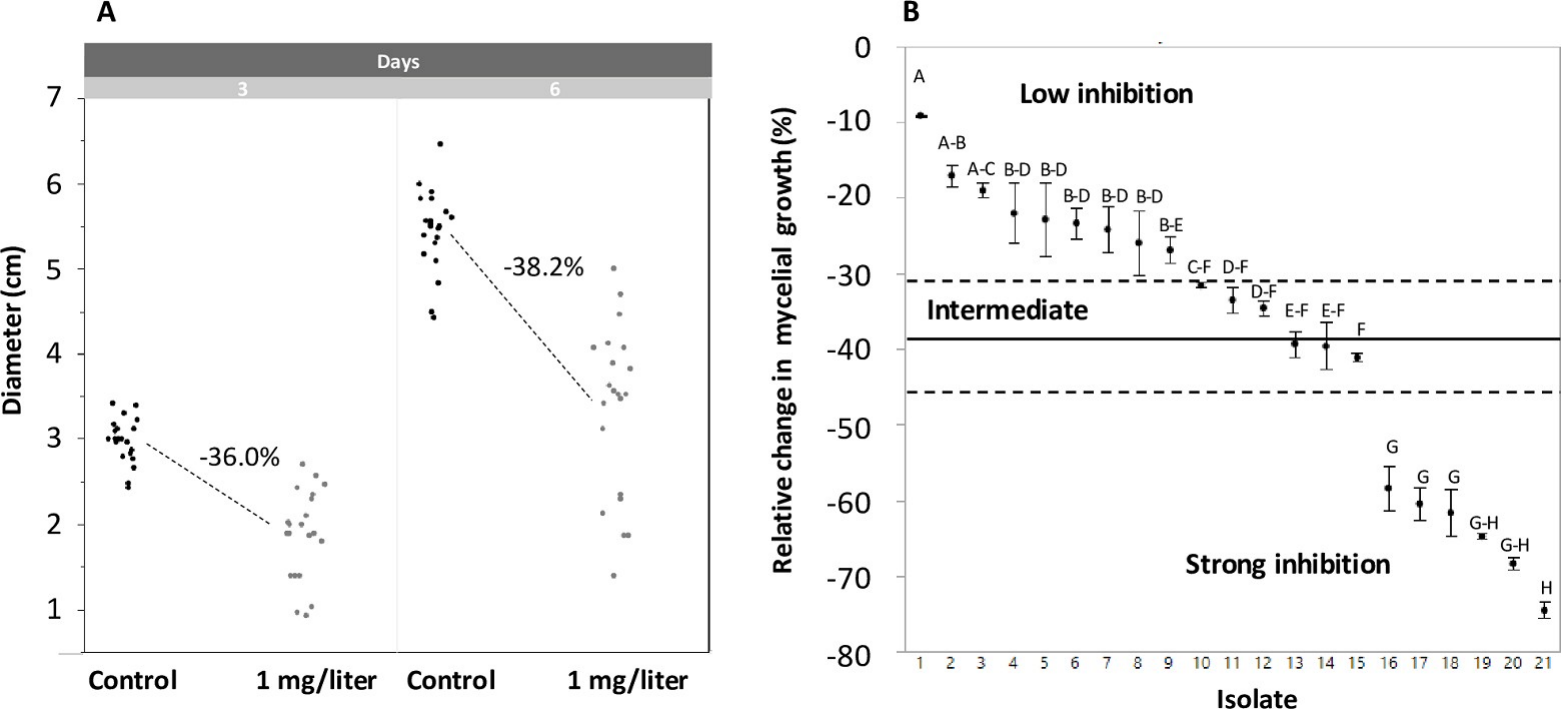
Effect of fungicide treatment on 21 isolates of *Fusarium fujikuroi*. A) effect on colony diameter at three and six days. B) variation among isolates for fungicide resistance as reduction of mycelial growth compared to the control, i.e. strong negative values indicate strong fungicide effect; the plot reports the results after six days. Bar: standard error of the mean. Isolate means not connected by the same letter are different (P<0.05) based on Tukey-Kramer HD test. Solid black line: mean; dotted lines: upper and lower Dunnet limit at for α = 0.05% based on the analysis of mean (ANOM).

The treatment with 1 mg/litre of prochloraz induced a reduction of colony size of 36.3% and 38.0% at three and six days, respectively (**Fig 10A**).

Differences among isolates in fungicide sensitivity were detected and were highly significant (P<0.0001) either after three days (d.f. = 20, S.S. = 2.01, F = 42.2, P<10^−4^) or six days (d.f. = 20, S.S. = 2.20, F = 66.01, P<10^−4^). Fungicide sensitivity measured at six days was strongly correlated with that measured at three days (r = 0.94; n = 21; P < 10^−5^). However, at six days there was more discrimination among isolates than at three days and differences were clear-cut (**Fig 10A**). Indeed, the most resistant isolate had a reduction in growth of 10% while the less resistant had a reduction of 75% (**Fig 10A and 10B**). Moreover, based on the combination of the Tukey-Kramer test and the analysis of mean (ANOM), it was apparent that groups with different fungicide sensitivity exist. Indeed, a group of six isolates was neatly separated from the remaining 15 and was well below the overall mean (**Fig 10B**). Among these, nine have a significant above-mean resistance, while six were in intermediate position.

### Association between molecular and biological features

The 33 isolates of *F*. *fujikuroi* considered for the pathogenicity test were representative of the SSR variation detected in the collection (**Fig 7**).

**Fig 11:**
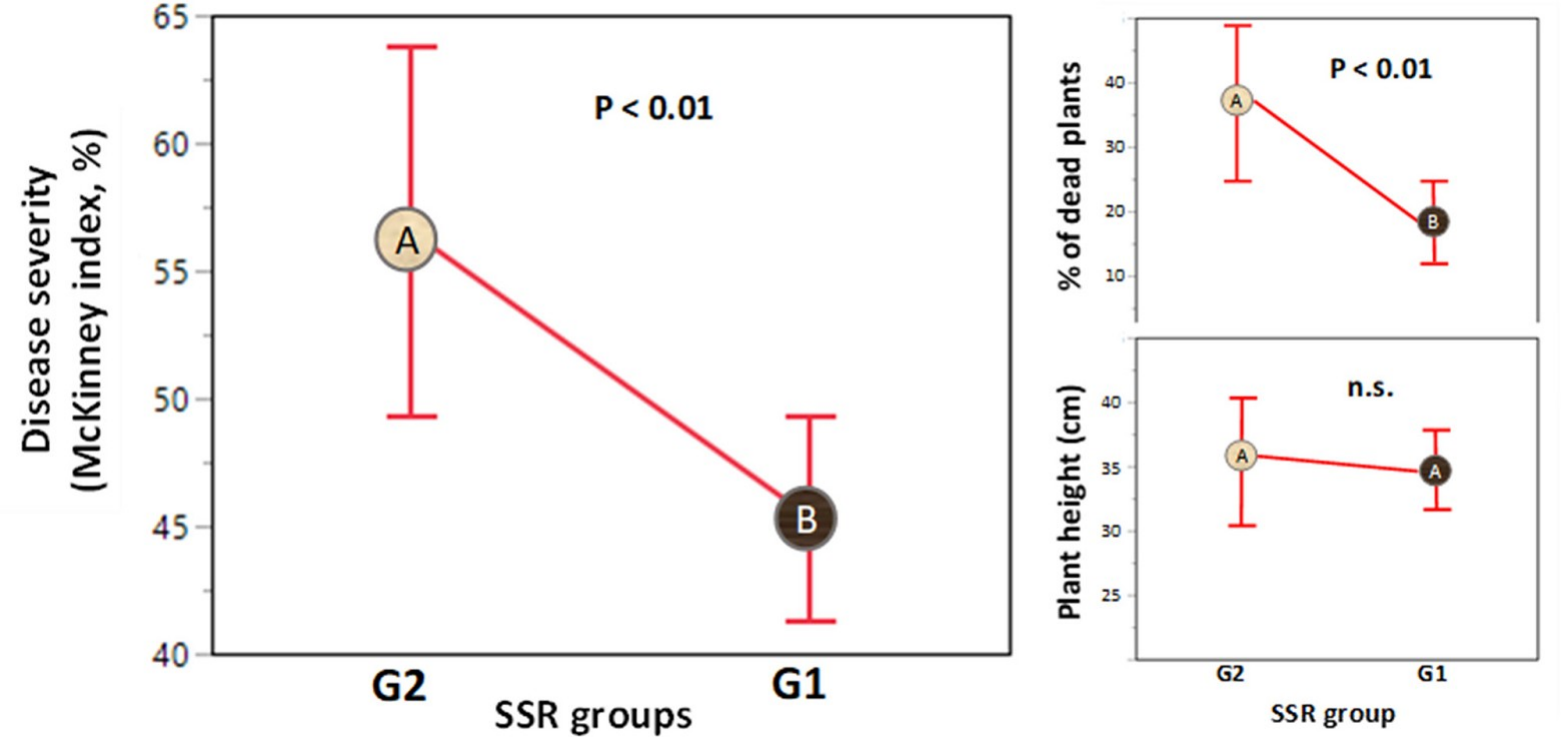
Comparison among the genetic groups G1 and G2 identified by Structure for disease severity (as summarized by McKinney Index). Small plots reported the results obtained for the percent of dead plants and the plant height separately. A different letter indicates significantly different means based on ANOVA.

Interestingly, the main genetic subdivision at K = 2 was associated with the variation in disease severity index (t test: P<0.01; **Fig 11**).

G1 was less virulent (McKinney index = 45.3%) compared with G2 (McKinney index = 56.5%; **Fig 11**).

Interestingly, when symptoms were considered separately, G1 and G2 were associated (t-test: P<0.01) with the ability to induce plant death but not (t-test: P = 0.37) with the severity of typical Bakanae (as measured by plant height; **Fig 11**). The two genetic groups did not show differences in mycelium growth rate or fungicide sensitivity. Finally, the two mating types did not differ for any of the biological features examined.

### Comparison between large samples of isolates from different countries and continents: Laos *vs* Italy

**Fig 12:**
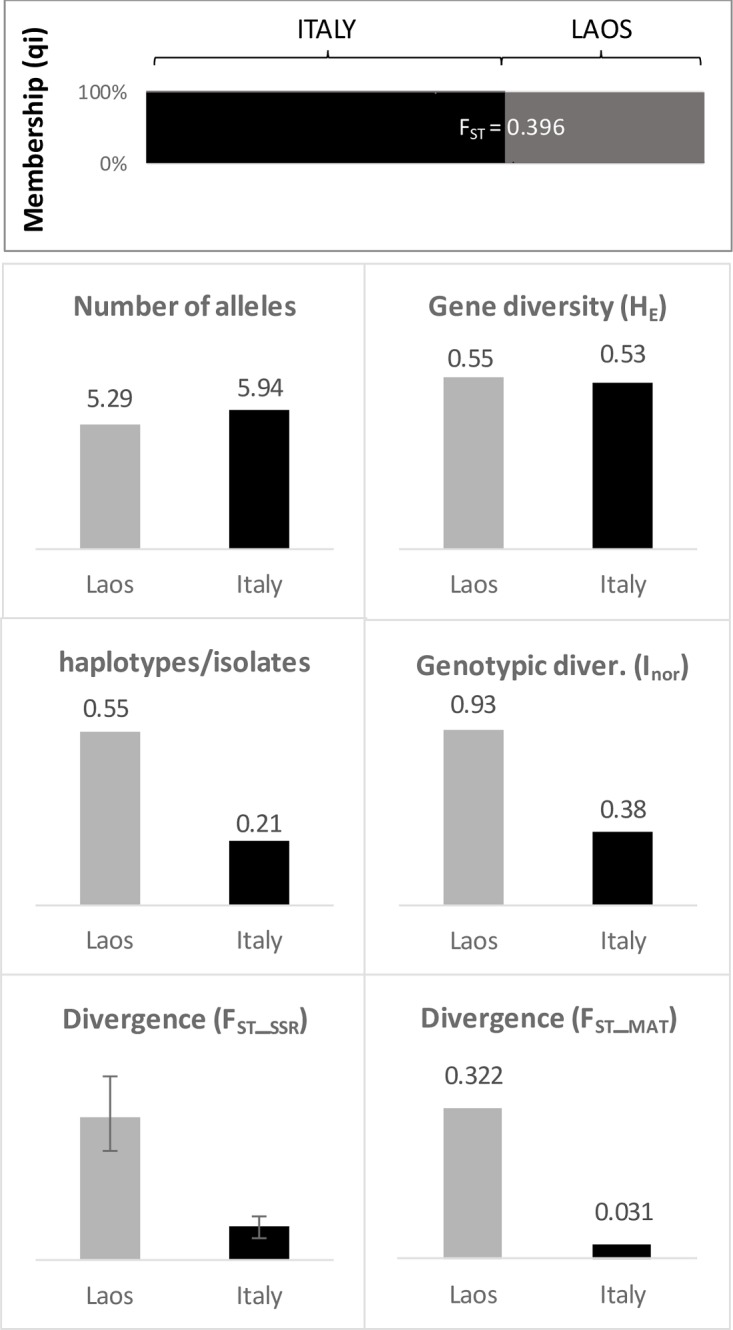
Comparison between Laos and Italy. The first panel on the top shows the results of cluster analysis by Structure with 315 Italian and 175 Laotian isolates. The other panels compare the two samples for various diversity statistics. In the divergence plots, F_ST_SSR_ = divergence among populations estimated based on microsatellite (SSR) marker frequencies. F_ST_MAT_ = divergence among populations estimated using mating types idiomorph frequencies.

Strong divergence among the Italian and the Laotian samples was found (F_ST_ = 0.396), with no shared haplotypes between the two countries. Isolates were attributed to the country of origin with very high membership values (q_i_ > 0.98 in all cases; **Fig 12**).

In **Fig 12** Italian and Laotian isolates are compared for several descriptive statistics. They showed a similar number of alleles and expected heterozygosity. In contrast, Laotian isolates showed higher haplotypes/isolates ratio (0.55 vs 0.20) and higher genotypic diversity (I_nor_; 0.93 vs 0.38) compared to the Italian isolates. The two countries showed striking differences (P < 0.001) for the level of population structuring which was more than 4-fold higher in Laos (F_ST_ = 0.237) than in Italy (F_ST_ = 0.056). The population divergence for the MAT locus reinforced this conclusion (0.322 and 0.031 in Laos and Italy, respectively).

**Fig 13:**
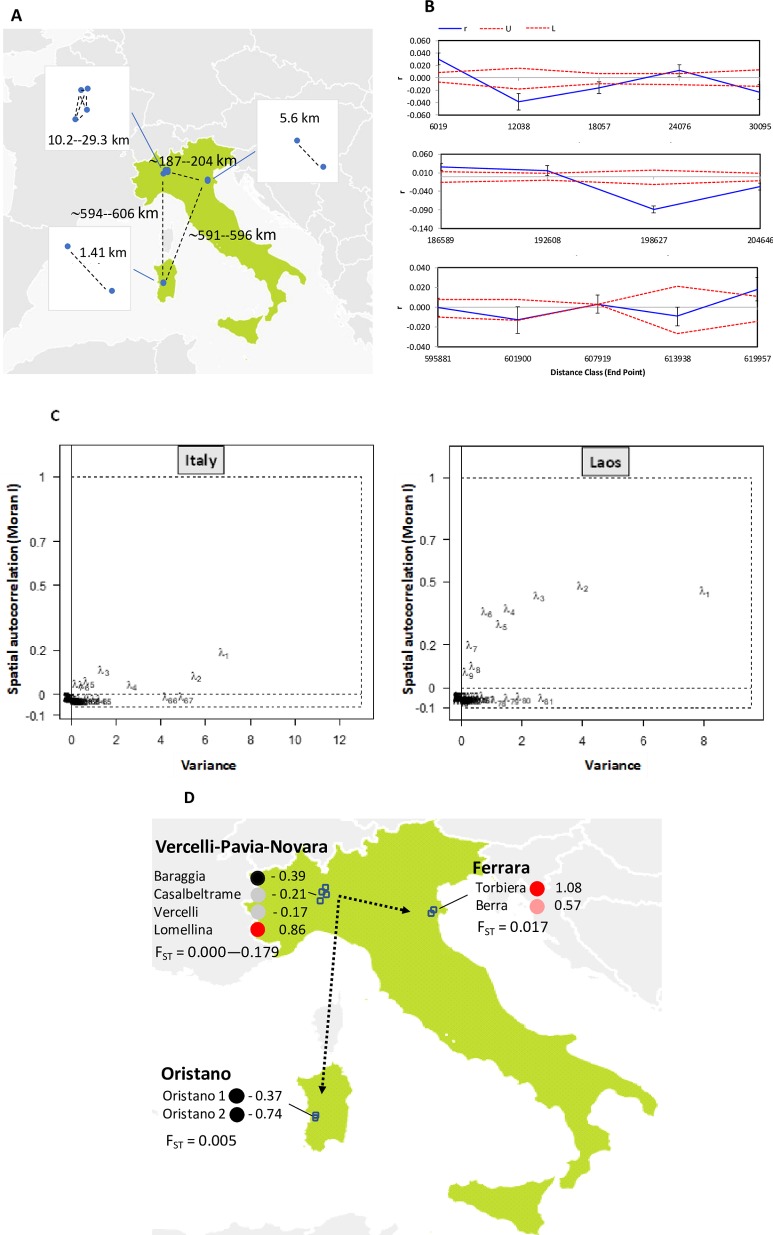
Comparison between Italy and Laos for the spatial structure. A) Distances among the Italian populations of *F*. *fujikuroi* analysed by Valente et al. (11). B) Results of spatial autocorrelation analysis. C) Scree plots of the spatial analysis of principal components (sPCA) showing the spatial and the variance components of the eigenvalues. D) Map of the first sPC scores. The range of pairwise population F_ST_ values for each region is reported. Dotted black arrows indicate the main possible directions of gene flow.

The spatial arrangement of the eight Italian populations is illustrated in **Fig 13A**. Compared to Laos, in Italy the genetic differences among isolates appeared less dependent on the geographical distances (**Fig 13B and 13C**).

Indeed, in Italy genetic similarity between isolates was higher than expected under the hypothesis of a random geographic distribution of genotypes in space either when low (<6 km) or high (187 Km) geographic distances were considered (**Fig 13B**) Similarly, isolates were more dissimilar than expected either at relatively low (12 Km) and at high distance (197 Km and 205 km). Moreover, several distance classes comprised in the range from 18 Km to 620 Km fitted the expectation of random geographic distribution of genetic variation.

sPCA analysis confirmed that the signal of spatial autocorrelation is weaker in Italy than in Laos (**Fig 13C**). For example, the first eigenvector in Italy had a Moran index (I) of 0.20, while this was 0.45 in Laos. Moreover, the first six eigenvectors in Laos had a higher Moran index (I) than the first eigenvector in Italy (**Fig 13C**). However, the results of global and local tests (**S9 Fig**) indicated the existence of positive spatial structure also for Italy. The map of the first sPCA (**Fig 13D**) indicated some tendency of the populations belonging to the same geographical areas to be genetically similar. Furthermore, the sPCA1 map also suggested that the main cultivation areas of rice in Italy (Vercelli-Novara-Pavia) retained more diversity and might be the source of inoculum of *F*. *fujikuroi* infesting other rice cultivation areas (Ferrara and Oristano).

## Discussion

An understanding of the population structures of plant pathogenic fungi can provide an insight into phylogenetic relationships and the biology of the organisms, including the mode of reproduction, migration, drift and selection [60, 65, 68, 69]. This ultimately improves our understanding of the evolutionary potential of pathogens and facilitates the development of strategies for plant breeding and improving the management of resistance genes [24]. In this study, we analysed a collection of *F*. *fujikuroi* from Lao PDR, a country in the region of rice domestication [15] and the origin of *Magnaporthe grisea* the causal agent of rice blast disease [16].

### Clonal propagation

The first key finding from this work is that there is a low probability that sexual or parasexual reproduction have prominent a role in shaping the genetic structure of the populations while in all probability clonal propagation is the prevalent mode of reproduction of *F*. *fujikuroi* in Laos. Several findings support this conclusion: a relevant fraction of the entire collection of isolates is represented by “true” clones, linkage disequilibrium (LD) is present in all populations and genetic groups; within populations and genetic groups, mating types are frequently skewed from 1:1 ratio, and genetic divergence between opposite mating types within the same population was detected.

Overrepresented genotypes and LD have been often considered suggestive of clonality [61]. In the present study, a simulation approach [63] indicated that most of the repeated genotypes did not arise by independent reproduction events but that they can instead be considered as belonging to the same clonal lineage. The multilocus LD persists within all populations even after removing clones. This allows us to reject the hypothesis that the Laotian populations have an “epidemic” population structure where the role of sex might have been “masked” [58]. Indeed, in this case, a limited number of frequent closely related genotypes originated from clonal reproduction is superimposed on a background population of relatively rare and unrelated genotypes from frequent sexual reproduction [58]. The persistence of LD after clone correction was also seen for eight populations in Italy [11] and for 14 populations in Taiwan [28] (**Table 8**). Thus, clone-corrected r_d_ values were significant in 32 populations of *F*. *fujikjuroi*, from quite different regions (Italy *versus* Laos and Taiwan) and analysed with different SSR markers (Italy and Laos *versus* Taiwan).

**Table 8:** Multilocus index of association (r_d_) for 32 geographical populations of *F*. *fujikuroi* across three countries (Laos, 10 populations; Italy = 8 populations; Taiwan = 14 populations). The r_d_ estimates were all for clone-corrected samples. Significance P values: *P<0.05, **P<0.01, P<0.001.

Laos^1^		Italy^2^		Taiwan^3^	
Population	r_d_	Population	r_d_	Population	r_d_
Pop 1	0.09***	Baraggia	0.18***	Taoyuan	0.44***
Pop 2	0.07***	Berra	0.35***	Hsinchu	0.20***
Pop 3	0.27***	Casal Beltrame	0.10**	Miaoli	0.19***
Pop 4	0.13***	Lomellina	0.10***	Taichung	0.13***
Pop 5	0.34***	Oristano 1	0.30***	Changhua	0.13***
Pop 6	0.19***	Oristano 2	0.32***	Yunlin	0.18***
Pop 7	0.56***	Torbiera di Codigoro	0.16***	Chiayi	0.32*
Pop 8	0.20***	Vercelli	0.21***	Tainan	0.18***
Pop 9	0.23***			Kaohsiung	0.12***
Pop 10	0.13***			Pingtung	0.15***
				Ilan	0.12***
				Nantou	0.34**
				Hualien	0.31***
				Taitung	0.20***
Mean	0.221		0.214		0.215
Dev.st	0.145		0.090		0.099

^1^This study.

^2^Calculated revisiting SSR data of ref. [11] using the same 17 SSR used in the present study.

^3^Reported in Table 2 (last column) of ref [28].

Specifically, the Laotian populations show clone-corrected r_d_ values from 0.07 to 0.56 (mean = 0.22) that are similar to those reported for Taiwan [28] (from 0.12 to 0.44; mean = 0.215) and Italy (from 0.09 to 0.323, mean = 0.214). In Taiwan, the observation of the sexual stage in the field and of high diversity at vegetative incompatibility (*vic*) loci indicate a significant role for sexual reproduction [28]. However, other evidences such as the presence of repeated genotypes, the observed pattern of r_d_ values, and the frequent bias from 1:1 MAT type ratio within populations clearly indicate non-random mating [28]. As possible causes of such non-random mating, the presence of female-sterile and poorly fertile individuals that might have an advantage during asexual propagation in the field and the limited movement of propagules in the field that might limit the match between opposite mating types are suggested [28].

The sexual stage of *F*. *fujikuroi*, has not been observed in the field in Italy [11] despite the occurrence of opposite MAT types within the population. Consequently, clonal reproduction is suggested as the mode of reproduction in Italy based on the high LD and the high clonal fraction [11]. Similarly, the sexual stage of *F*. *fujikuroi* has not been observed in Laos (Burgess, personal communication). This does not necessarily imply that sexual reproduction is absent in Laos but suggests that it is probably not frequent.

Non-zero r_d_ values can also be explained by slow asymptotic decay of historic disequilibrium. This would imply that all populations were founded very recently, which seems unlikely, and that recombination does not have time to operate. Alternatively, it can be hypothesised that populations are ancient but over thousands of generations the recombination events were rare. Laotian populations showed strong LD also when only unlinked (or loosely linked) loci were considered; additionally, LD persisted after accounting for population structure. These indicate long-range LD and high background LD in the analysed populations, which is consistent with rare recombination. Finally, another cause of non-zero r_d_ values can be the epistatic selection; however, it seems unlikely that this prompted frequent LD among unlinked loci in all the populations examined in Laos.

Mating-type frequencies determine the probability that two random isolates will be compatible. Thus, if sexual reproduction is operating and MAT genes are functional, the approximate 1:1 ratio between mating types can be maintained by negative-frequency-dependent selection, a kind of balancing selection [70, 64]. In this case, the mating-type ratio can be “buffered” against any strong variation from the 1:1 ratio (e.g. [65]). Additionally, under this circumstance genetic divergence between mating types is prevented. However, in this study we observed a strong bias from the 1:1 ratio in several populations and overall, that again suggests that sexual reproduction is rare in Laos. Moreover, the evidence of genetic divergence (of restriction to gene flow) between opposite MAT idiomorphs co-occurring within the same population further corroborated this inference.

### Founder effect and spatial structure

The observed uneven distribution of mating types within populations can also be explained by demographic effects. For example, the annual cropping cycles and possibly burning of the crop residues may mean that fungal plant-pathogen populations in a crop are often founded by a small fraction of the total population [71, 64]. This is well illustrated by the study on *F*. *fujikuroi* of Carter et al. [25] in California where the near fixation of the MAT-1 idiomorph was documented (observed ratio 170 MAT-1: 4 MAT-2).

Alternatively, if the mating-type idiomorphs (or closely linked loci) differ in viability or clonal fecundity, the mating-type ratio might deviate significantly from the 1:1 null hypothesis [72], even though the population may be entirely sexual [73, 74].

The spatial analysis of genetic variation indicates that migration of *F*. *fujikuroi* is restricted overall and that genetic drift and founder effects play an important role in the evolution of this fungus. This is indicated by the presence of moderate genetic divergence among geographical populations, the low but significant genetic divergence between geographical areas that tends to correspond to separate patches, and the general tendency of neighbour isolates of being more similar than expected compared to a random distribution. The presence of spatial structure and isolation-by-distance was also observed in Taiwan [28]. However, we also observed that in some cases populations collected far apart can be genetically similar; this indicates the potential for long-range migration too, most likely by human-mediated dispersal (exchange) of infested seeds, or infested rice straw which is used for a range of purposes that may result in its movement over some distance.

To gain more insight on this aspect, in this study, and for the first time in *F*. *fujikuroi*, we compared the results obtained with the same marker set in two agricultural systems with contrasting characteristics: low input, rainfed and traditional (Laos PDR) *vs* high input irrigated and modern (Italy). In Italy, the level of population structuring was much lower than in Laos and the majority of populations showed a mating type ratio not skewed from 1:1. This might reflect higher gene-flow in Italy than in Laos. It is well known that in Italy seed exchange is common among rice cultivation areas and that the geographic area comprised by the provinces of Vercelli, Pavia and Novara can be considered as the “hub” of seed exchanges in Italy. Intriguingly, the genetic spatial pattern of *F*. *fujikuroi* in Italy seems to reflect this reality. Although the provinces of Vercelli, Pavia and Novara are the most important in term of total cultivated area and rice production, the most suitable area for rice seed multiplication is Oristano province, in Sardinia Island, where more favourable environmental conditions minimise disease incidence and lead to higher seed quality.

In contrast in Lao, movement of rice seed between the three areas sampled in this project would have been restricted until more recent times, as they remained somewhat isolated until reasonable roads were built by French colonial administration to facilitate trade and people movement. Thus, movement would have been restricted until more recent decades when roads were modernised, modern vehicles facilitated more efficient road transport. These recent developments also coincided with expanding efforts on the breeding of more modern cultivars and their distribution.

The findings reported in this paper provide further evidence that the evolutionary potential of this pathogen not only depends on its intrinsic characteristics but is likely to be strongly influenced by other external factors, most likely by the dynamics of (infested) seed exchange. McDonald and Linde [24], and Meng et al. [75] have postulated that human mediated gene flow can increase the adaptive potential of a fungal pathogen such as *F*. *fujikuroi*. Consequently, quarantine regulations and seed treatments may be required to reduce population connectivity and the evolutionary potential of the fungus.

The direct comparison between Laos and Italy also reveals evidence of the possibility of an abrupt genetic variation likely due to a strong founder effect at high geographical scale. Indeed, when the 175 Laotian and the 334 Italian isolates were compared, a clear-cut genetic divergence was observed (F_ST_ = 0.396) without any shared haplotypes. Moreover, Laotian and Italian isolates showed different fungicide sensitivity (**S10 Fig**).

### Association between SSR population structure and pathogenicity

We found that *F*. *fujikuroi* isolates in Laos were split into two main well-defined genetic clusters which have a non-random spatial distribution and were associated with different levels of pathogenicity. This might reflect a founder effect. Indeed, even though small-holder family farming production systems prevail in most rice-growing areas of Laos[17], disease incidence increased recently due to changes in agricultural practices, an effect that can probably be attributed to the introduction of newer varieties [18]. Alternatively, this might be due to the co-evolutionary history between host and pathogen populations. Indeed, the two SSR genetic groups of *F*. *fujikuroi* differed in their ability to induce seedling death but not for Bakanae symptoms. Thus, differences among SSR groups of *F*. *fujikuroi* may have arisen because of differential selective pressure exerted by the host on the pathogen. This hypothesis would imply that host rice populations have a different degree of resistance against the isolates inducing seedling death but not against the isolates inducing Bakanae, i.e. the host resistance genes against isolates inducing different symptoms are not the same. This would imply that in the *O*. *sativa-F*. *fujikoroi* plant-pathosystem co-evolution of closely related pathogens interacting with the same host could lead to different adaptive outcomes. This possibility has been recently shown for *Hordeum vulgare-Pyrenophora teres* in a landrace metapopulation [69]. However, dedicated cross- inoculation studies are necessary to shed light on this question for *O*. *sativa-F*. *fujikoroi* plant-pathosystem.

Interestingly, an analysis of field isolates conducted by Niehaus et al. [76] provided evidence of a strong correlation between the pathotype (Bakanae or stunting/foot rot), and the ability to produce either gibberellic acid or fumonisins. The phylogenetic analyses revealed two subclades of *F*. *fujikuroi* strains according to their pathotype and secondary metabolite profiles [76]. However, a large difference in secondary metabolite production capability between G1 and G2 groups as the Niehaus phylogenetic classes seems unlikely. Although the two main genetic groups (G1 and G2) showed a quantitative difference with respect to pathogenicity, most of the tested Laotian isolates of *F*. *fujikuroi* have the potential to both cause elongation as well as also browning and stem rot in the crown region and seedlings death. This was also observed for Italian isolates [11]. Additionally, our study also showed that isolates recovered from stunted/rotted plants are not more virulent than those collected from plants showing the typical Bakanae symptoms (abnormal elongation). However, a direct comparison between our findings and those of Niehaus and co-workers is difficult. Indeed, the study of Niehaus and co-workers includes seed and seedling inoculation tests. In comparison our pathogenicity tests only involved a seed inoculation method. Thus, the symptoms observed in the two experiments might not be comparable. Further research is needed to understand if the genetic groups identified in this work subtend phylogenetic entities or, more likely, might be considered a case of variation among populations within the same *taxon*. Indeed, the population structure of *F*. *fujikuroi* associated with Bakanae on rice can be complex as concluded by Choi et al. [77] in Korea.

## Supporting information

S1 Table Gene diversity (H_E_) by SSR locus and for each geographical population of *F*. *fujikuroi*. For each SSR the chromosome position is given. Populations are sorted from North to South.
(PDF)

S2 Table Effect of closely linked loci on the index of association (r_d_) when all isolates or clone corrected sample were considered. (PDF)

S3 Table Number and percentage of digenic disequilibria (LD) within the 10 populations *of F*. *fujikuroi*. Linkage disequilibrium was evaluated for all markers as also distinguishing between linked and unlinked loci pairs. Two loci were considered in LD if P<0.05 after 10^5^ randomizations.
(PDF)

S1 Fig Relationships among the ten geographical populations of *F*. *fujikuroi*. A) pairwise F_ST_ matrix based on AMOVA. B) Neighbor-joining tree. The optimal tree with the sum of branch length = 0.254 is shown. The tree is drawn to scale, with branch lengths in the same units as those of the evolutionary distances used to infer the phylogenetic tree (F_ST_). The analysis was conducted in MEGA X.
(TIF)

S2 Fig Results of Structure analysis after clone-correction. Upper scatterplot: mean of the estimated of Ln probability of the data, lnP(K), as a function of the number of assumed populations, K. Lower scatterplot on the left: determination of the “true” number of populations based on the method of Evanno [44]; lower histogram on the right: results of individuals to population assignment at K = 2. Isolates were sorted based on their coefficient of membership (q_i_) for G1 in descending order and irrespective of the geographical population of origin.
(TIF)

S3 Fig Results of DAPC cross-validation. Proportion of successful outcome prediction = proportion of isolates of the validation dataset (10% of the total dataset) that is correctly assigned to the genetic groups. Number of PCA axes retained: number of principal components used to run DAPC (and to build the discriminant functions) using the training dataset (90% of the total dataset). Individual replicates appear as points, and the density of those points in different regions of the plot is displayed in blue (30 replicates). Top panel: validation procedure from 10 to 70 PCA axes (step = 10 axes); bottom panel = validation procedure from 10 to 30 (step = 1 axe). The plain and dashed lines indicate the mean expectation from a random classifier, and its 95% confidence interval.
(TIF)

S4 Fig DAPC analysis: assignment of the 175 isolates of *F*. *fujikuroi* to the four clusters identified by *snapclust*. Each row represents an isolate. Heat colors represent membership probabilities (red = 1, white = 0); blue crosses represent the prior cluster provided to DAPC (in this context the four clusters identified by *snapclust*). DAPC retrieves the clusters identified by *snapclust* (blue crosses are on red rectangles).
(TIF)

S5 Fig Representation of the first, the second, and the third discriminant functions of the discriminant analysis of principal components (DAPC) carried out on the four groups found using the *snapclust* analysis. (TIF)

S6 Fig Results of spatial principal component analysis (sPCA). A) Plot of the sPCA eigenvalues. B) Spatial and variance components of the sPCA eigenvalues (λ). Results of Monte Carlo simulations to test (C) the presence of positive spatial autocorrelation (global test), or negative spatial autocorrelation local structures (D) in the genetic data.
(TIF)

S7 Fig Results of spatial principal component analysis. (A) Results of Monte Carlo simulation to test the significance of spatial autocorrelation for the second spatial principal component (sPC2). B) Average sPC2 scores for each of the ten populations of *F*. *fujikuroi* (population are sorted from North to South). Blank, grey and black dots: strongly negative, intermediate and highly positive average sPC2 scores, respectively C) sPC2 scores as a function of the coefficient of membership (q_i_) returned by Structure at K = 2. G1 and G2: the two groups identified by Structure. D1-D4: the four clusters identified by *snapclust*/DAPC.
(TIF)

S8 Fig Interaction between isolates mycelial growth (diameter of the colony, cm) and time (days). Each line represents a different isolate (number of tested isolates = 21).
(TIF)

S9 Fig Results of the global and local test for the Italian isolates of *F*. *fujikuroi*. (TIF)

S10 Fig Results of fungicide treatments (1 mg/L of Procholoraz after six days) on colony growth (diameter, cm). (TIF)
