# Supplementary material for: Clonality, spatial structure, and pathogenic variation in Fusarium fujikuroi from rain-fed rice in southern Laos
Source: PLoS One. 2019 Dec 23;14(12):e0226556. doi: 10.1371/journal.pone.0226556 (PMC6927642; doi:10.1371/journal.pone.0226556)
Supplement: S1 Table — For each SSR the chromosome position is given. Populations are sorted from North to South. (PDF) [file pone.0226556.s001.pdf]

S1 Table. Gene diversity (H_E) by SSR locus and for each geographical population of *F. fujikuroi*. For each SSR the chromosome position is given. Populations are sorted from North to South.

		Gene diversity (H_E)										
**Locus**	**Chromosome**	**position**	**Pop1**	**Pop2**	**Pop3**	**Pop4**	**Pop5**	**Pop6**	**Pop7**	**Pop8**	**Pop9**	**Pop10**
TUZ1 (CTG)	chr1	1219282	0.17	0.08	0.23	0.00	0.00	0.00	0.00	0.00	0.00	0.11
TUZ7 (TTC)	chr1	1725346	0.64	0.87	0.44	0.54	0.65	0.18	0.22	0.22	0.77	0.45
TUZ2 (GAA)	chr1	1801650	0.45	0.24	0.40	0.46	0.53	0.00	0.23	0.23	0.41	0.35
TUZ11 (TG)	chr2	653156	0.50	0.53	0.63	0.53	0.66	0.18	0.22	0.22	0.49	0.60
TUZ10 (ACTGCC)	chr2	3471775	0.79	0.74	0.78	0.58	0.66	0.47	0.21	0.21	0.70	0.49
TUZ13 (GACCAA)	chr2	4042535	0.66	0.55	0.64	0.54	0.59	0.55	0.22	0.22	0.60	0.44
TUZ16 (TCT)	chr3	2410839	0.66	0.16	0.67	0.00	0.00	0.00	0.00	0.00	0.00	0.00
TUZ33 (CAGA)	chr3	2795949	0.24	0.43	0.23	0.00	0.00	0.00	0.00	0.00	0.11	0.11
SSR2 (CTAC)	chr3	3608020	0.47	0.49	0.53	0.26	0.13	0.00	0.11	0.11	0.35	0.35
SSR26 (CAA)	chr3	3799327	0.36	0.52	0.00	0.36	0.51	0.00	0.21	0.21	0.43	0.20
TUZ14 (CAAGC)	chr4	908441	0.36	0.63	0.13	0.50	0.53	0.44	0.21	0.21	0.49	0.21
SSR8 (CT)	chr4	1998834	0.78	0.81	0.61	0.69	0.73	0.35	0.31	0.31	0.44	0.65
TUZ21 (GT)	chr5	1975918	0.55	0.61	0.53	0.44	0.63	0.18	0.62	0.62	0.49	0.53
CR6.1 (AAAG)	chr6	2426661	0.82	0.73	0.76	0.63	0.65	0.64	0.22	0.22	0.60	0.46
CR7.1 (GA)(GT)	chr7	1860620	0.79	0.70	0.57	0.63	0.63	0.44	0.11	0.11	0.60	0.29
CR8.1 (TATG)	chr8	1186241	0.82	0.72	0.73	0.27	0.62	0.00	0.22	0.22	0.58	0.65
CR9.2 (CGCAA)	chr9	1214911	0.66	0.63	0.54	0.67	0.13	0.44	0.22	0.22	0.62	0.20
