# Supplementary material for: Clonality, spatial structure, and pathogenic variation in Fusarium fujikuroi from rain-fed rice in southern Laos
Source: PLoS One. 2019 Dec 23;14(12):e0226556. doi: 10.1371/journal.pone.0226556 (PMC6927642; doi:10.1371/journal.pone.0226556)
Supplement: S2 Table — (PDF) [file pone.0226556.s002.pdf]

S2 Table. Effect of closely linked loci on the index of association (r_d) when all isolates or clone corrected sample were considered. Significance level: ***P<0.001

	All markers (as in Table 6)		Without markers at < 200 kb	
**Population**	**All isolates**	**Clone corrected**	**All isolates**	**Clone corrected**
Pop1	0.12***	0.09***	0.11***	0.08***
Pop2	0.08***	0.07***	0.08***	0.07***
Pop3	0.350***	0.27***	0.35***	0.32***
Pop4	0.34***	0.13***	0.33***	0.11***
Pop5	0.47***	0.34***	0.49***	0.34***
Pop6	0.21***	0.19***	0.24***	0.24***
Pop7	0.64***	0.56***	0.62***	0.55***
Pop8	0.32***	0.20***	0.33***	0.20***
Pop9	0.28***	0.23***	0.29***	0.24***
Pop10	0.37***	0.13***	0.40***	0.14***
Mean	0.32	0.22	0.32	0.23
