# Supplementary material for: Clonality, spatial structure, and pathogenic variation in Fusarium fujikuroi from rain-fed rice in southern Laos
Source: PLoS One. 2019 Dec 23;14(12):e0226556. doi: 10.1371/journal.pone.0226556 (PMC6927642; doi:10.1371/journal.pone.0226556)
Supplement: S3 Table — Linkage disequilibrium was evaluated for all markers as also distinguishing between linked and unlinked loci pairs. Two loci were considered in LD if P<0.05 after 105 randomizations. (PDF) [file pone.0226556.s003.pdf]

S3 Table. Number and percentage of digenic disequilibria (LD) within the 10 populations *of F. fujikuroi*. Linkage disequilibrium was evaluated for all markers as also distinguishing between linked and unlinked loci pairs. Two loci were considered in LD if P<0.05 after 10^5 randomizations.

		ALL SSR markers			Linked SSR markers			Unlinked SSR markers		
Population	N. of isolates	N. of pairs	N. of pairs in LD	% of pairs in LD	N. of pairs	N. of pairs in LD	% of pairs in LD	N. of pairs	N. of pairs in LD	% of pairs in LD
Pop1	23	136	64	47.1	13	2	15.4	123	62	50.4
Pop2	24	136	36	26.5	13	4	30.8	123	32	26.0
Pop3	16	120	86	71.7	10	7	70.0	110	79	71.8
Pop4	14	91	49	53.8	6	3	50.0	85	46	54.1
Pop5	15	91	67	73.6	6	5	83.3	85	62	72.9
Pop6	11	45	10	22.2	4	1	25.0	41	9	22.0
Pop7	18	105	57	54.3	6	3	50.0	99	54	54.5
Pop8	16	91	58	63.7	6	3	50.0	85	55	64.7
Pop9	19	91	65	71.4	6	3	50.0	85	62	72.9
Pop10	19	120	70	58.3	10	5	50.0	110	65	59.1
Mean	17.5	102.6	56.2	54.3	8.0	3.6	47.4	94.6	52.6	54.9
