# Supplementary figures and images for: Clonality, spatial structure, and pathogenic variation in Fusarium fujikuroi from rain-fed rice in southern Laos
Source: PLoS One. 2019 Dec 23;14(12):e0226556. doi: 10.1371/journal.pone.0226556 (PMC6927642; doi:10.1371/journal.pone.0226556)

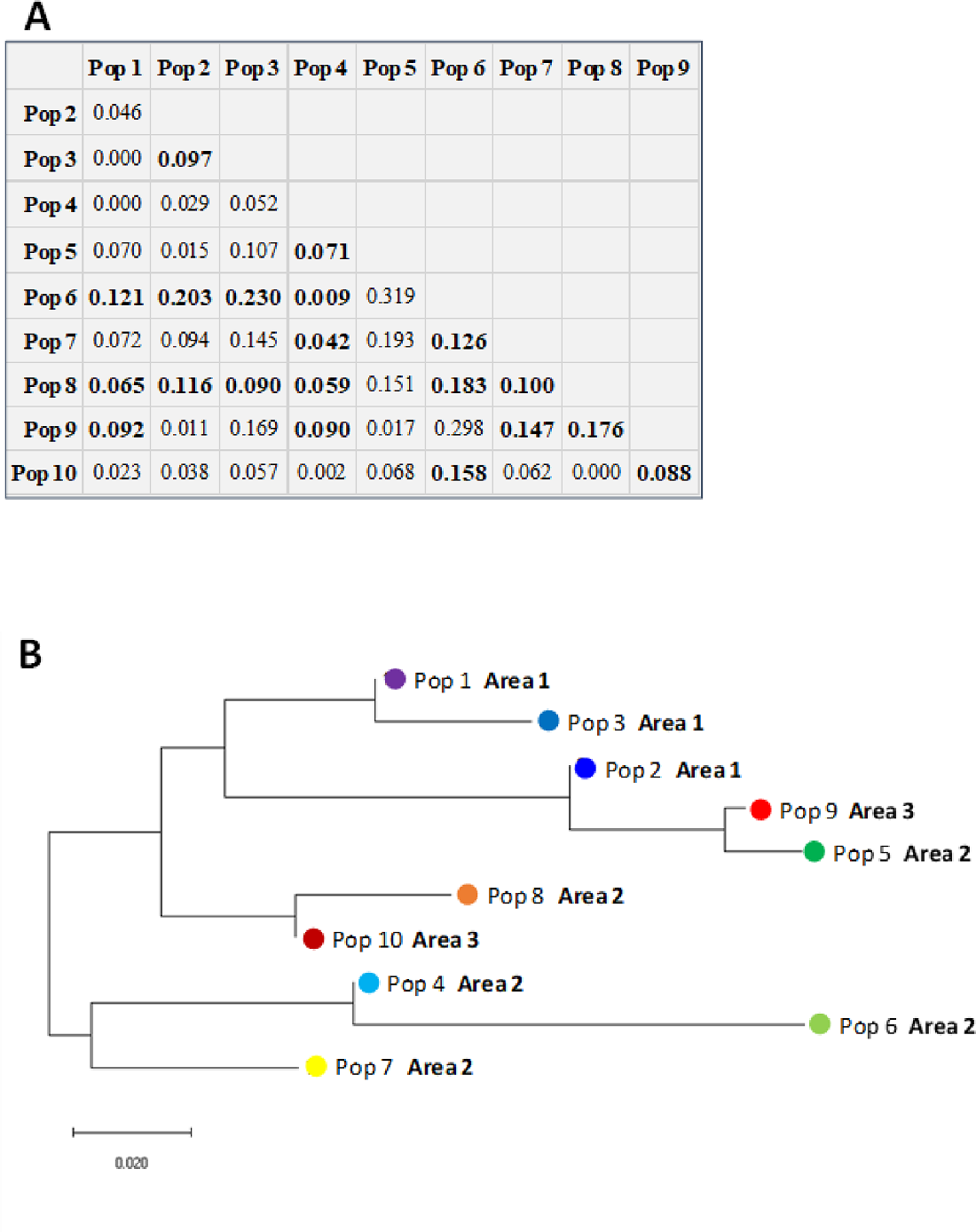

Supplement: S1 Fig — A) pairwise FST matrix based on AMOVA. B) Neighbor-joining tree. The optimal tree with the sum of branch length = 0.254 is shown. The tree is drawn to scale, with branch lengths in the same units as those of the evolutionary distances used to infer the phylogenetic tree (FST). The analysis was conducted in MEGA X. (TIF) [file pone.0226556.s004.tif]

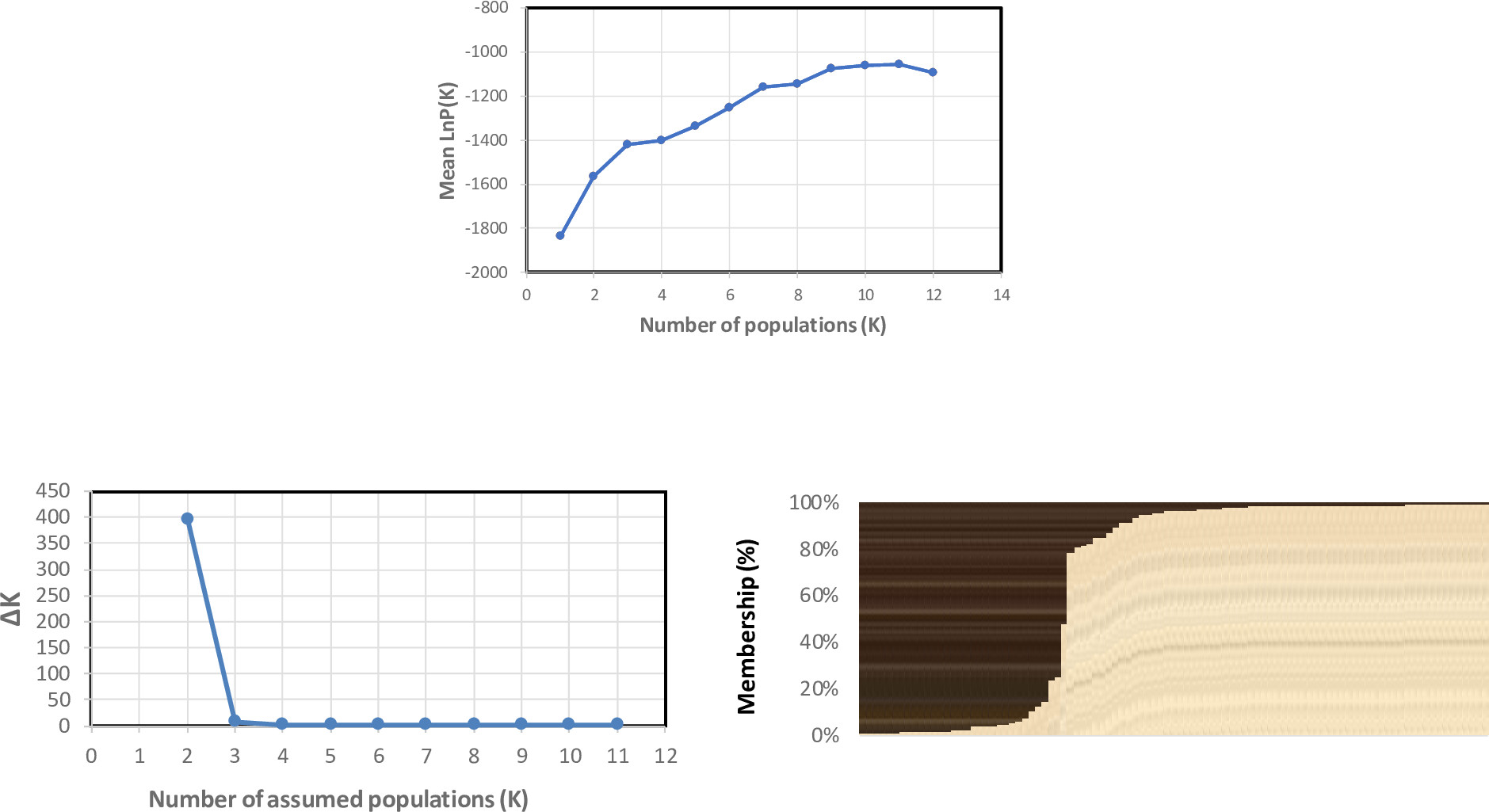

Supplement: S2 Fig — Upper scatterplot: mean of the estimated of Ln probability of the data, lnP(K), as a function of the number of assumed populations, K. Lower scatterplot on the left: determination of the “true” number of populations based on the method of Evanno [44]; lower histogram on the right: results of individuals to population assignment at K = 2. Isolates were sorted based on their coefficient of membership (qi) for G1 in descending order and irrespective of the geographical population of origin. (TIF) [file pone.0226556.s005.tif]

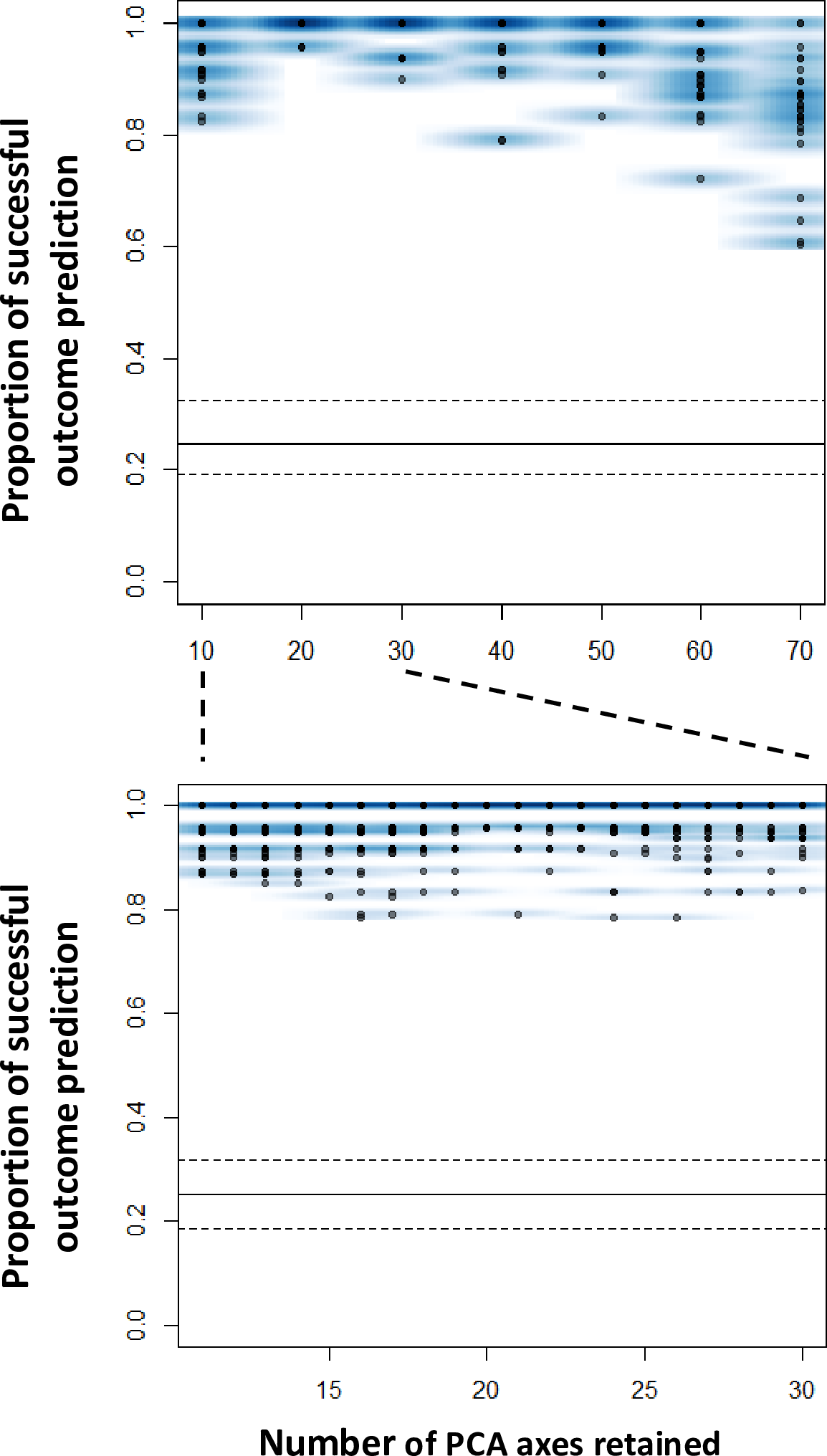

Supplement: S3 Fig — Proportion of successful outcome prediction = proportion of isolates of the validation dataset (10% of the total dataset) that is correctly assigned to the genetic groups. Number of PCA axes retained: number of principal components used to run DAPC (and to build the discriminant functions) using the training dataset (90% of the total dataset). Individual replicates appear as points, and the density of those points in different regions of the plot is displayed in blue (30 replicates). Top panel: validation procedure from 10 to 70 PCA axes (step = 10 axes); bottom panel = validation procedure from 10 to 30 (step = 1 axe). The plain and dashed lines indicate the mean expectation from a random classifier, and its 95% confidence interval. (TIF) [file pone.0226556.s006.tif]

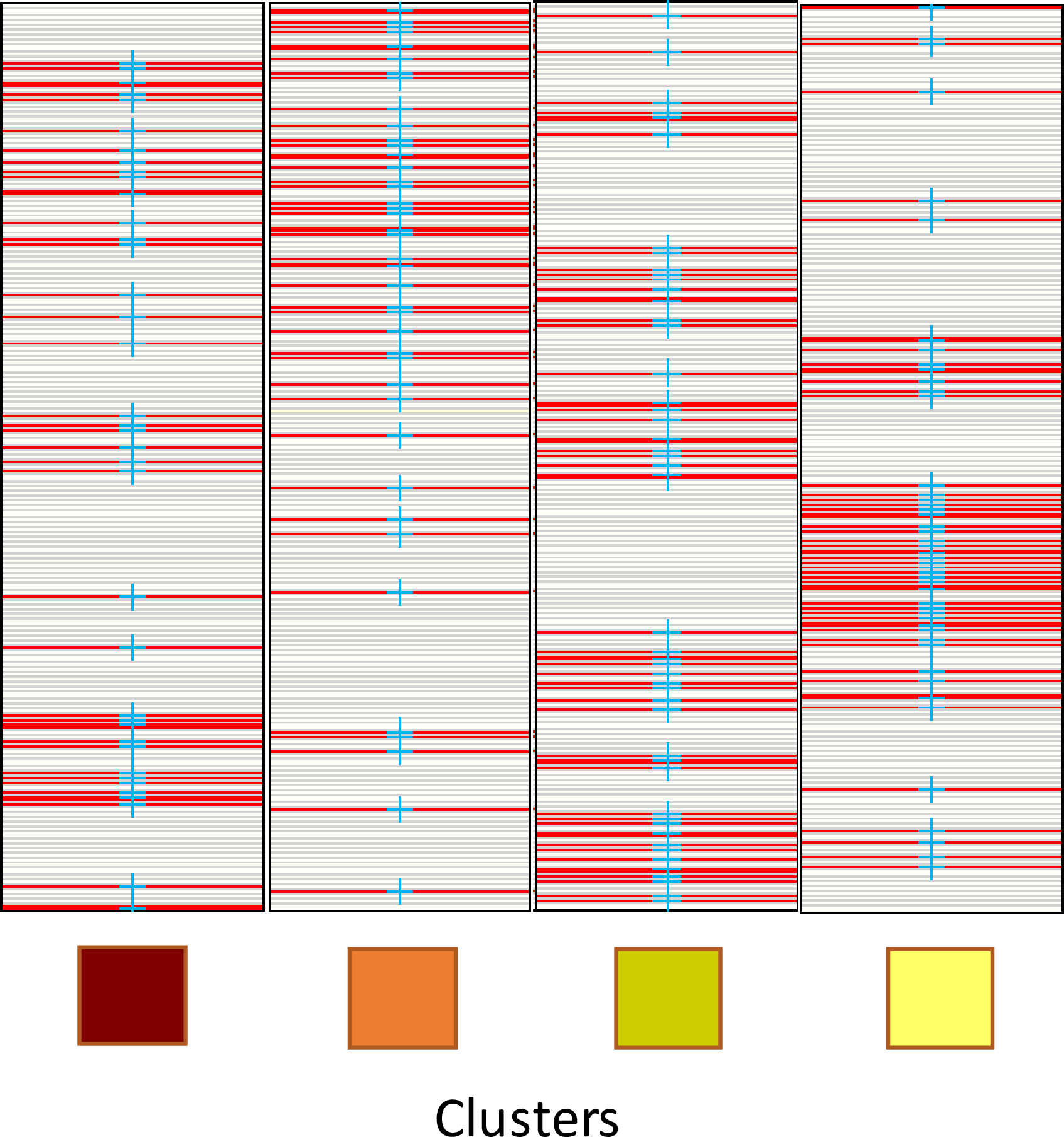

Supplement: S4 Fig — Each row represents an isolate. Heat colors represent membership probabilities (red = 1, white = 0); blue crosses represent the prior cluster provided to DAPC (in this context the four clusters identified by snapclust). DAPC retrieves the clusters identified by snapclust (blue crosses are on red rectangles). (TIF) [file pone.0226556.s007.tif]

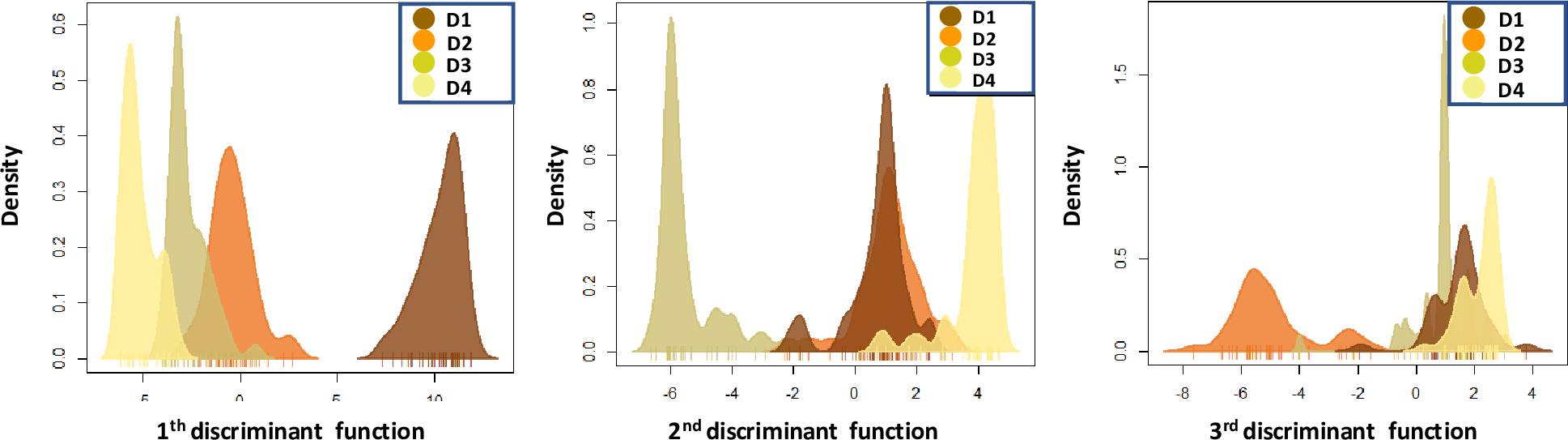

Supplement: S5 Fig — (TIF) [file pone.0226556.s008.tif]

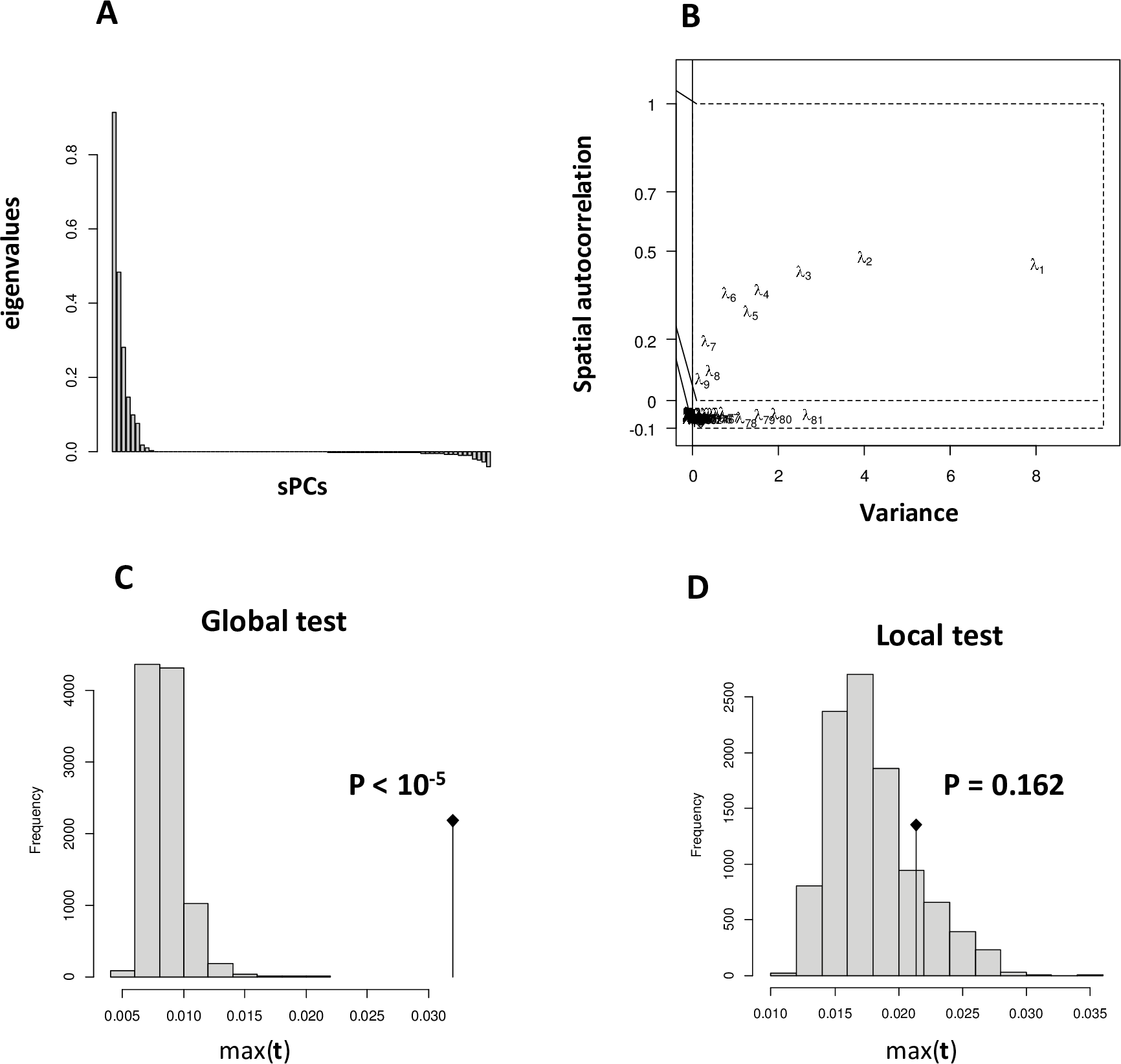

Supplement: S6 Fig — A) Plot of the sPCA eigenvalues. B) Spatial and variance components of the sPCA eigenvalues (λ). Results of Monte Carlo simulations to test (C) the presence of positive spatial autocorrelation (global test), or negative spatial autocorrelation local structures (D) in the genetic data. (TIF) [file pone.0226556.s009.tif]

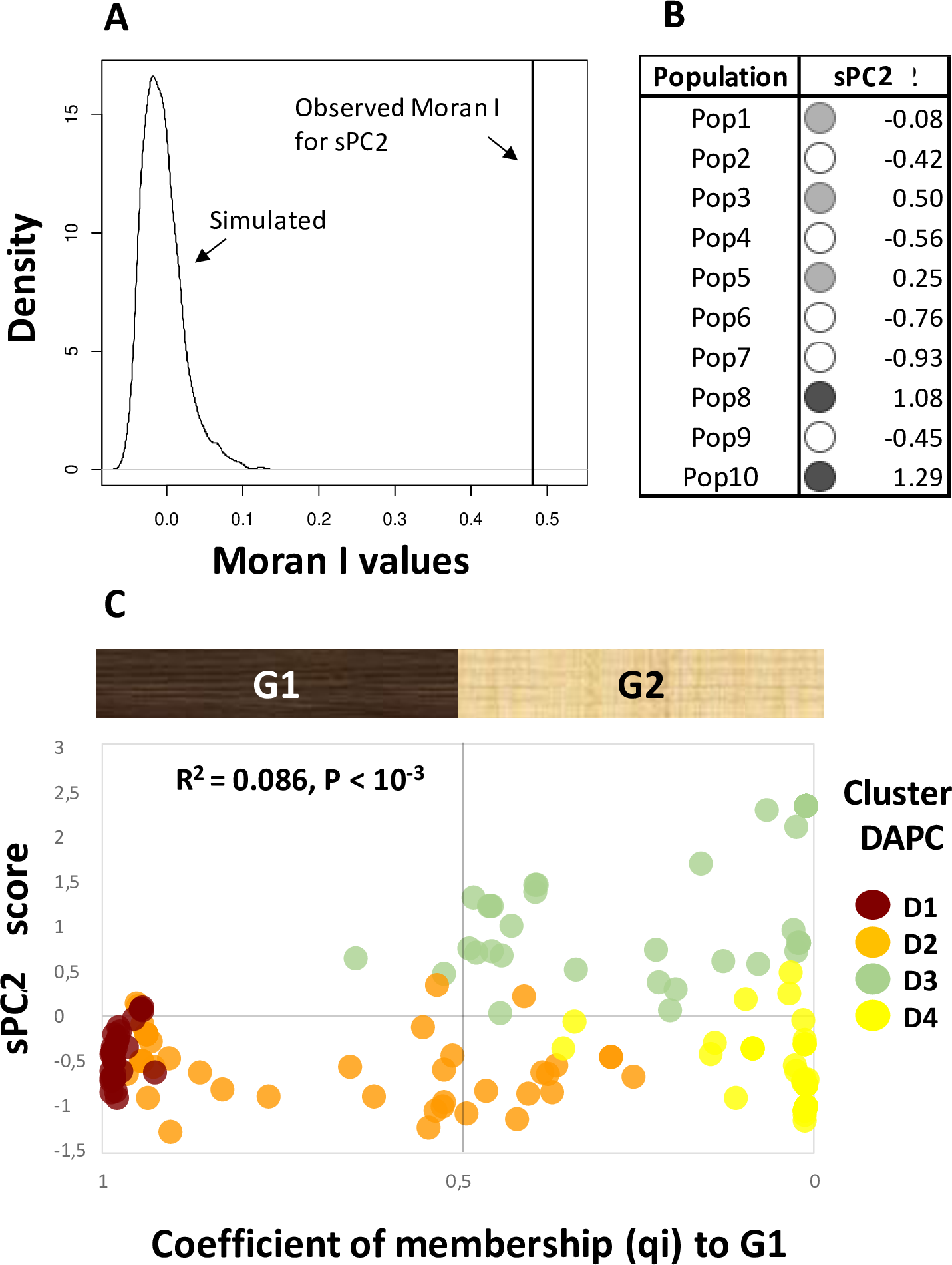

Supplement: S7 Fig — (A) Results of Monte Carlo simulation to test the significance of spatial autocorrelation for the second spatial principal component (sPC2). B) Average sPC2 scores for each of the ten populations of F. fujikuroi (population are sorted from North to South). Blank, grey and black dots: strongly negative, intermediate and highly positive average sPC2 scores, respectively C) sPC2 scores as a function of the coefficient of membership (qi) returned by Structure at K = 2. G1 and G2: the two groups identified by Structure. D1-D4: the four clusters identified by snapclust/DAPC. (TIF) [file pone.0226556.s010.tif]

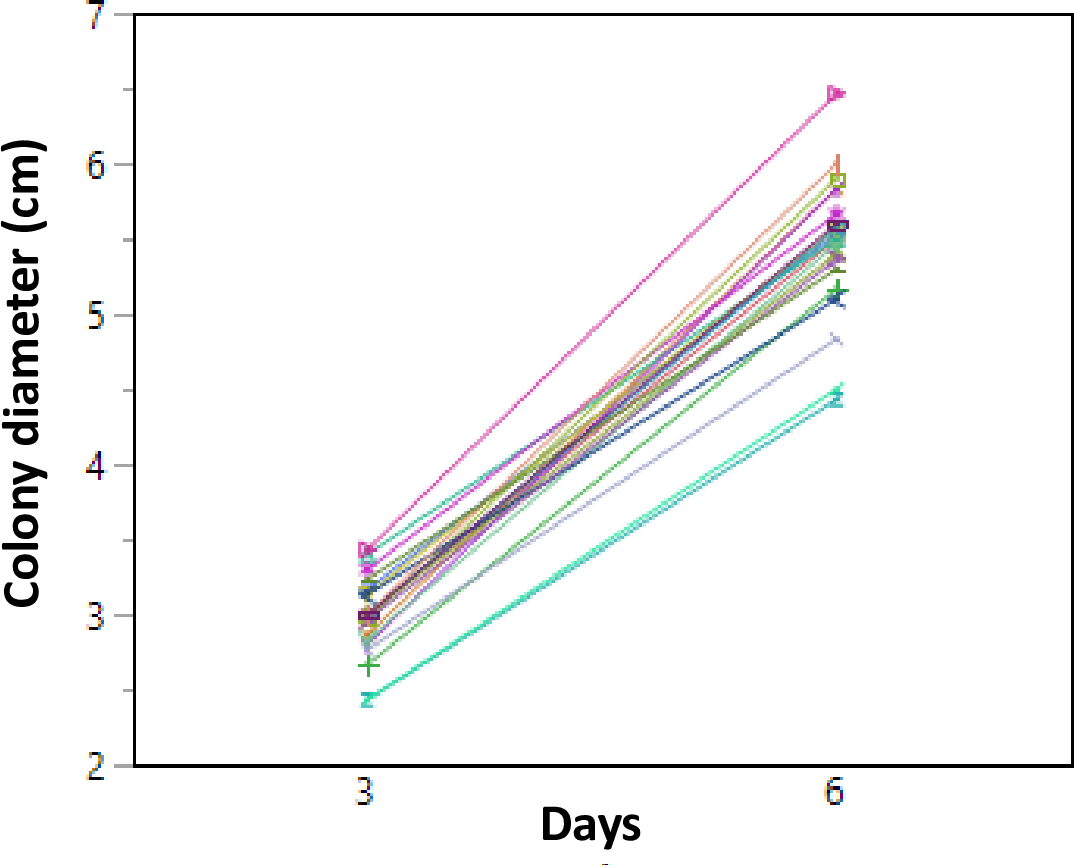

Supplement: S8 Fig — Each line represents a different isolate (number of tested isolates = 21). (TIF) [file pone.0226556.s011.tif]

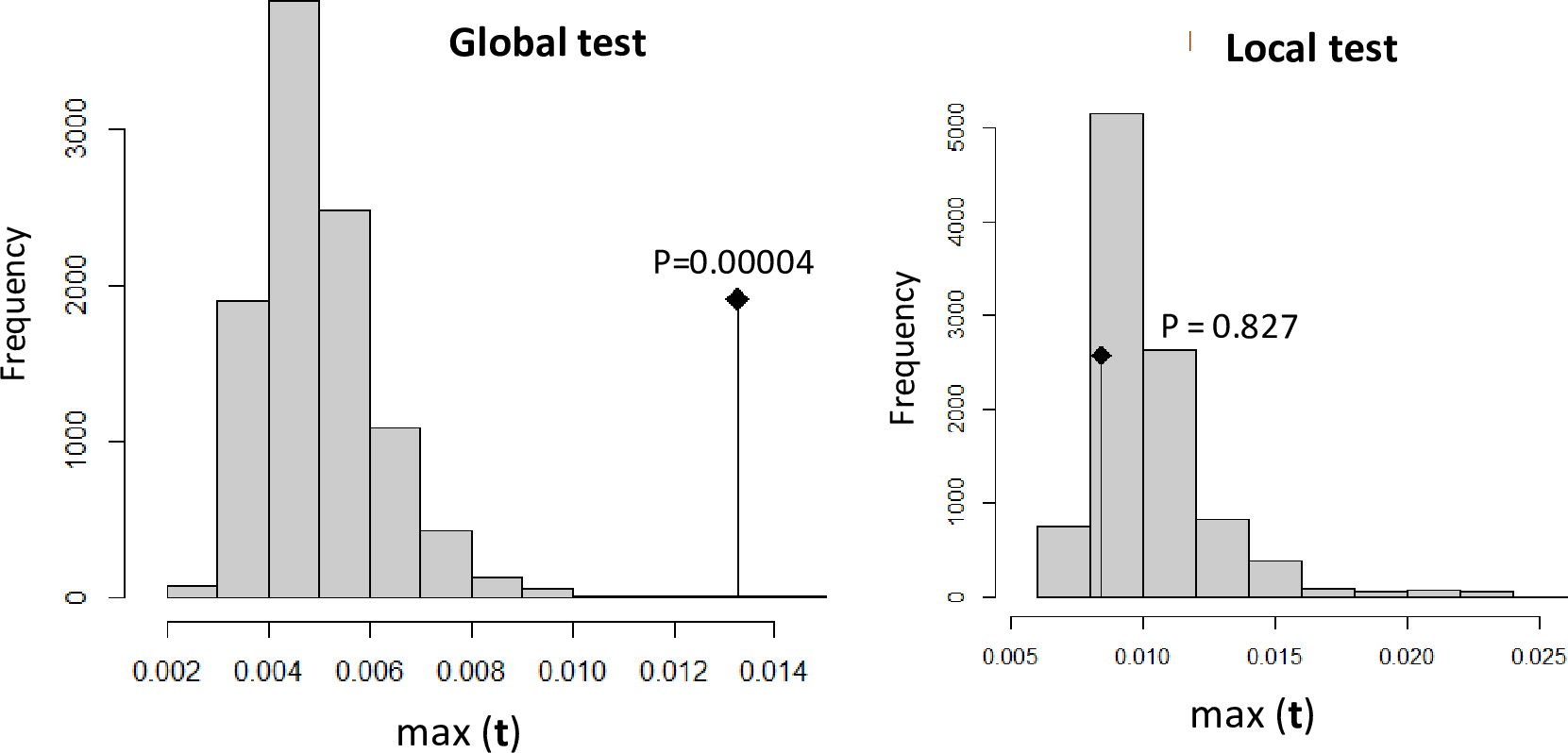

Supplement: S9 Fig — (TIF) [file pone.0226556.s012.tif]

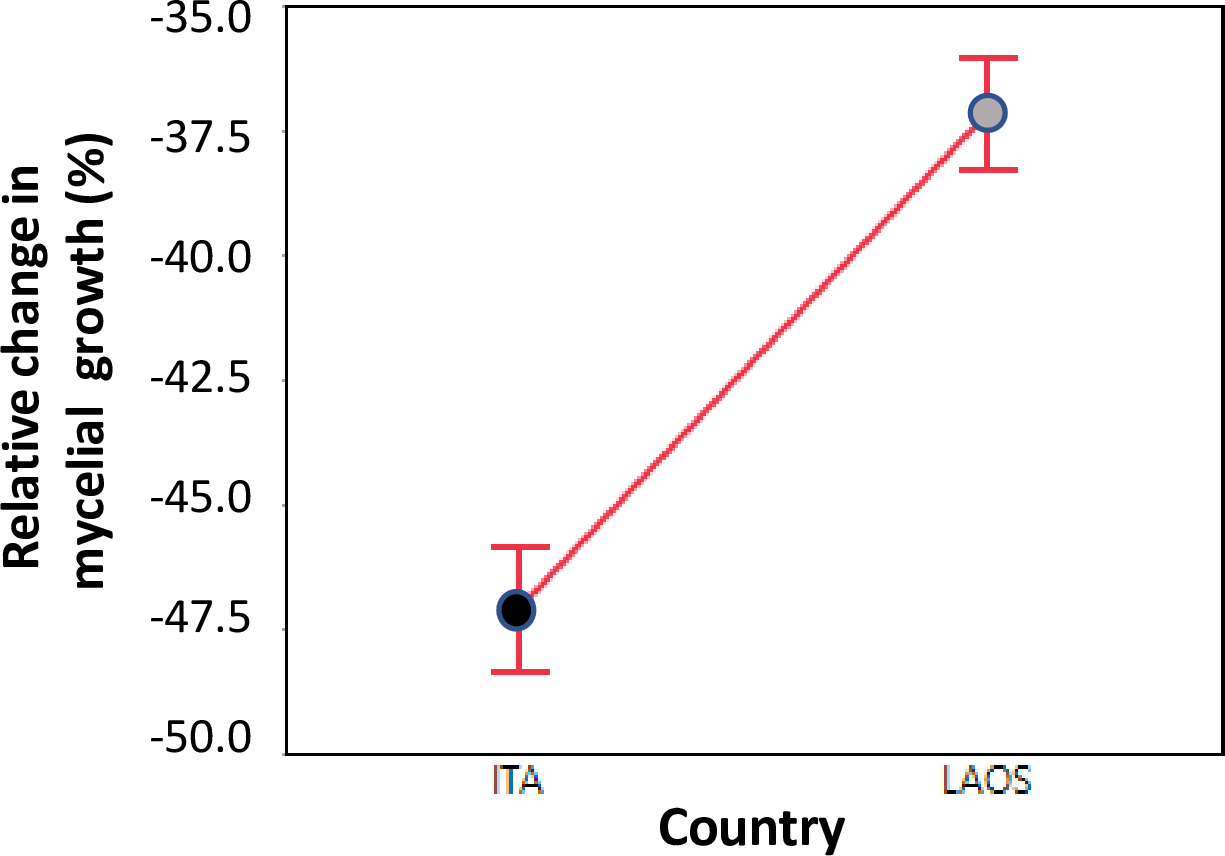

Supplement: S10 Fig — (TIF) [file pone.0226556.s013.tif]
